# Paradoxical roles of ATF6α and ATF6β in modulating disease severity caused by mutations in collagen X

**DOI:** 10.1016/j.matbio.2018.03.004

**Published:** 2018-09

**Authors:** M. Forouhan, K. Mori, R.P. Boot-Handford

**Affiliations:** a Wellcome Trust Centre for Cell-Matrix Research, Faculty of Biology, Medicine and Health, Manchester Academic Health Science Centre, Manchester, UK; b Department of Biophysics, Graduate School of Science, Kyoto University, Kyoto, Japan

## Abstract

Whilst the role of ATF6α in modulating the unfolded protein response (UPR) has been well documented, the function of its paralogue ATF6β is less well understood. Using knockdown in cell culture and gene ablation in mice we have directly compared the roles of ATF6α & β in responding to the increased ER stress induced by mutant forms of type X collagen that cause the ER stress-associated metaphyseal chondrodysplasia type Schmid (MCDS). ATF6α more efficiently deals with the disease-associated ER stress in the absence of ATF6β and conversely, ATF6β is less effective in the absence of ATF6α. Furthermore, disease severity in vivo is increased by ATF6α ablation and decreased by ATF6β ablation. In addition, novel functions for each paralogue are described including an ATF6β-specific role in controlling growth plate chondrocyte proliferation. The clear demonstration of the intimate relationship of the two ATF6 isoforms and how ATF6β can moderate the activity of ATF6α and vice versa is of great significance for understanding the UPR mechanism. The activities of both ATF6 isoforms and their separate roles need consideration when deciding how to target increased ER stress as a means of treating MCDS and other ER stress-associated diseases.

## Highlights

• ATF6α more efficiently controls ER stress in the absence of ATF6β
• ATF6α ablation increases disease severity in the MCDS mouse
• ATF6β knock out diminishes the pathological changes in the MCDS mouse
• ATF6β plays role in controlling growth plate chondrocyte proliferation
• Indeed, ablation of ATF6α increases disease severity in the MCDS mouse whereas ablation of ATF6β diminishes the pathological changes.
• In addition, a number of new and distinct roles for each isomer were identified.
• The relationship of the ATF6 isoforms and how they moderate each other's activity is of great significance for understanding the UPR mechanism in general.
• Both their interactions and their distinct roles need consideration when deciding how to target ATF6 pathways as a means of treating MCDS and other related ER stress-associated diseases.

## Introduction

The normal homeostasis of the endoplasmic reticulum (ER) depends on maintaining a balance between the entry of the newly synthesised proteins into the ER, their folding, the transit of correctly folded proteins to the Golgi, and the degradation of misfolded/unfolded proteins. There are a number of different insults, including expression of a mutant protein, which can compromise this balance and cause ER-stress. The elevated ER-stress is characterised by accumulation of misfolded/unfolded proteins within the ER lumen. Eukaryotic cells cope with ER stress by activating a cascade of intracellular signalling pathways collectively known as the unfolded protein response (UPR). The classical UPR is mediated through three ER resident transmembrane proteins; IRE1, PERK, and ATF6. These UPR transducers are bound to the chaperone BiP in their inactive state but as ER- stress increases, BiP is sequestered into the ER lumen by interaction with accumulating unfolded proteins. Loss of BiP binding leads to activation of the stress sensors which attempt to alleviate ER stress and restore ER homeostasis in terms of protein folding. Activation of the stress sensors i) enhances the ER-folding capacity by up- regulation of chaperones such as BiP/GRP78; ii) reduces the nascent protein load in the ER lumen by PERK-dependent attenuation of general protein synthesis; and iii) stimulates ER- associated degradation (ERAD) by which misfolded proteins are retrotraslocated to the cytoplasm, ubiquitinated and then degraded by the proteasome [[1], [2], [3], [4]].

Whilst the UPR is primarily an adaptive mechanism to accommodate fluctuations in ER stress caused by physiological/environmental changes in demand for protein folding in the ER, its prolonged activation has been linked to the pathology of many diseases including metaphyseal chondrodysplasia type Schmid (MCDS), a mild dwarfism caused by mutations in the gene encoding type X collagen [5]. The UPR response in MCDS allows survival of chondrocytes, but it alters the differentiation of hypertrophic chondrocytes and ultimately impairs bone growth and results in an expanded cartilage growth plate hypertrophic zone (HZ) [[6], [7], [8]]. Transcriptome analysis of the growth plate HZ in a knock-in mouse model of MCDS carrying the *Col10a1* p.N617K MCDS-causing mutation revealed increased ER stress and a robust UPR characterised by activation of all three canonical ER stress sensors, Atf6, Perk, and Ire1 in response to the intracellular retention of mutated and misfolded collagen X protein within the hypertrophic chondrocytes [[6], [7], [8]]. The role of Ire1/Xbp1 signalling pathway in MCDS pathology was shown to be of little biological importance, as chondrocyte-specific ablation of *Xbp1* in MCDS mice (Col10a1 p.N617K) did not alter the disease severity in MCDS [9]. The role of Atf6 and Perk signalling pathways in the MCDS disease mechanism remained to be identified. Here we used in vitro and in vivo approaches to investigate the role of the Atf6 branch of the UPR in the MCDS disease mechanism. ATF6 has two isoforms, ATF6α and ATF6β. Both isoforms are ubiquitously expressed ER-localised basic leucine zipper (bZIP) transcription factors consisting of an N-terminal cytoplasmic domain with transcriptional activity, a transmembrane segment, and an ER luminal domain that senses ER-stress [[10], [11], [12]].

In addition to structural similarities, ATF6α and ATF6β also share the same mechanism of activation under ER stress, which is translocation from ER to the Golgi apparatus where they are sequentially cleaved at their luminal and transmembrane domains with Golgi- resident proteases S1P and S2P, resulting in liberation from the ER membrane of their cytosolic N-terminal regions, designated as ATF6α(N) and ATF6β(N) [10,11,13]. These cleaved and activated forms subsequently migrate to the nucleus where they bind as homo- or heterodimers to the promoter of genes containing ER stress response elements (ERSE-I and -II), originally identified in the genes encoding the ER chaperones such as BiP, and induce transcription [10,11,[14], [15], [16], [17]].

Previous work has established that there is a functional redundancy between two isoforms of ATF6 as a single knockout of each ATF6 isoform in mice or fish was without phenotypic effect in the absence of exogenous ER stress whereas their double deficiency was embryonically lethal [[18], [19], [20]]. Despite the presence of an overlapping and compensatory function between isoforms of Atf6, there is some evidence for isoforms having distinct functions which are not redundant and only become apparent under conditions of increased ER stress. For instance, Atf6α ko mice develop liver steatosis when challenged with tunicamycin whereas Atf6β ko mice do not [21,22]. Also, ATF6β, but not ATF6α, has been shown to have a specific role in the regulation of the *PIGF* and *Wfs1* genes [23,24]. Studies on each isoform have indicated that ATF6α is inducible by increases in ER stress whereas ATF6β is not [18,[25], [26], [27]]. In addition, ATF6α appears to have a higher transcriptional activity than ATF6β [25,26].

Given that ATF6β, like ATF6α, senses and becomes activated in response to the accumulation of unfolded/misfolded proteins within the ER, it would be logical to expect activation of ATF6β in MCDS. However, only induction and activation of ATF6α has been studied to date [6,7]. Here we make the first direct and systematic comparison of the roles of ATF6α and -β under ER stress caused by the expression of a variety of MCDS-causing collagen X mutations in our cell culture model of the disease activated UPR [28]. In addition, we crossed *Atf6*α and *Atf6*β knockout mice with *Col10a1* p.N617K mouse model of MCDS to investigate the role of each isoform in the MCDS disease mechanism in vivo. We demonstrate that Atf6β, like Atf6α, is involved in the MCDS disease mechanism. The two isoforms of Atf6 play some distinct roles in modulating the MCDS-mediated ER stress but, also work in concert to finely tune the extent and magnitude of Atf6-mediated UPR response. Our results also reveal a beneficial role for Atf6α but a detrimental role for Atf6β, in modulating the disease severity in MCDS and an indispensable role for Atf6β in controlling chondrocyte proliferation in the murine growth plate.

## Results

### Loss of ATF6α but not ATF6β compromises the cells ability to respond to increased ER stress induced by the expression of mutant collagen X

MCDS pathology arises as a direct result of ER stress caused by intracellular retention of mutated and misfolded collagen X protein [7,8]. To determine the role of ATF6 in this process, we first used an in vitro approach to examine the consequences of *ATF6*α and β knockdown on the level of ER stress induced in our MCDS cell culture models (HeLa cells transiently expressing one of four different MCDS-causing mutant forms of collagen X protein). Consistent with previous studies [6,7], *ATF6*α mRNA was induced in cultures expressing each of the MCDS mutant forms of collagen X tested (S1a Fig.). In addition, *ATF6*β mRNA was also mildly induced by the expression of each of the MCDS constructs compared to un-transfected (UTF) cells (S1b Fig.). The expression of both *ATF6*α and *ATF6*β mRNAs were efficiently and selectively knocked down/silenced in all four MCDS mutant-expressing cell cultures co-transfected with the respective siRNAs (S1a–b Fig.). These changes in *ATF6*α & β mRNAs were also apparent at the protein level (S1c–f Fig.) confirming the relative efficiency of the knockdown protocol (see Materials and methods).

**Fig. 1:**
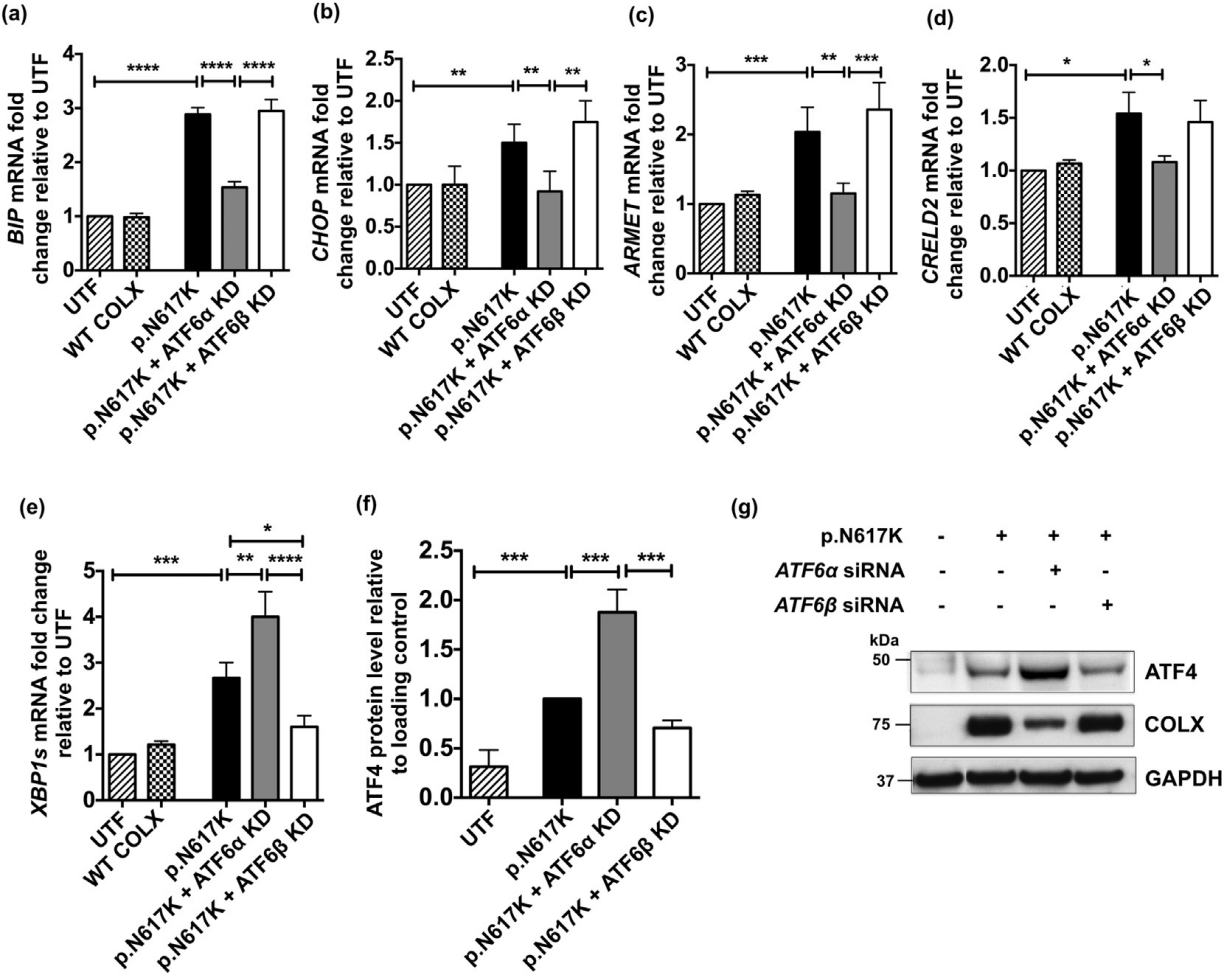
Effects of *ATF6*α and *ATF6*β knockdown in cells expressing p.N617K MCDS-causing mutant form of collagen X protein. The expression of either *ATF6*α or *ATF6*β was knocked-down in HeLa cells using siRNAs. These cells were then transiently transfected with expression constructs encoding either the wild type collagen X or p.N617K mutant form of the protein. 48 h post transfection, (a) RNA was extracted and analysed with real time qPCR for the expression of (a) *BIP*, (b) *CHOP*, (c) ARMET and (d) CRELD2 and (e) the spliced XBP1, XBP1s. Mean ± SEM (n = 5). *p < 0.05, **p < 0.01, ***p < 0.001, ****p < 0.0001. Untransfected cells (UTF) served as controls. (f) Cell lysates were extracted and immunoblotted using an anti- ATF4 antibody. The level of ATF4 protein was quantified relative to GAPDH loading control. Values represent Mean ± SEM from three independent experiments. ***p < 0.001, ****p < 0.0001 as determined by ANOVA. UTF: Untransfected cells. Typical western blots for ATF4 and collagen X proteins in *ATF6*α or *ATF6*β siRNA-mediated knocked down cells expressing N671K.

We next tested the ability of these MCDS mutant-expressing cells to induce ER stress- associated genes that are, at least in part, ATF6-dependant in the absence (or marked reduction) of each isoform of ATF6. The results for the Col10a1p.N617 K construct are presented in Fig. 1 and discussed below; results for the other 3 constructs (NC1del10, p.Y598D & p.G618V) are presented in S2 Fig, S3 Fig Figs. The expression of p.N617K induced *BiP* and *CHOP* mRNA (Fig. 1a & b) and also induced the chaperones *ARMET* and *CRELD2* (Fig. 1c & d). The knockdown of *ATF6*α prevented the induction of *BiP*, *CHOP ARMET and CRELD2* mRNAs in cells expressing p.N617K whereas the knockdown of *ATF6*β had little to no effect on the expression of these mRNAs (Fig. 1a–d). Similar results for the effects of *ATF6*α & β with one or two minor differences were obtained when expressing the 3 other MCDS-causing collagen X mutations (S2 Fig.).

These results demonstrate that, as expected based on previous work, *ATF6*α (but not *ATF6*β) plays a key role controlling *BiP* and *CHOP* induction in response to the ER stress induced by the expression of mutant collagen X. *ATF6*α also plays a role in the induction of *ARMET* mRNA whereas a role for *ATF6*β is less clear cut. *CRELD2* induction appeared to be *ATF6*α dependent only in the context of the ER stress induced by the p.N617K mutation (Fig. 1d and S2 Fig. d).

### Knockdown of *ATF6*β suppresses MCDS-induced IRE1 pathway activity whereas knockdown of *ATF6*α stimulates the PERK pathway

Although *BiP* and *CHOP* mRNAs are often used as crude indicators of the level of ER stress in a system, this association is lost when you interfere with the expression of major regulators of their expression such as ATF6 as described above. In order to gain insight into the effects of ATF6 knockdown on the overall levels of ER stress experienced by cells expressing the mutant forms of collagen X, the activities of the IRE1 and PERK pathways were assessed by measuring the levels of spliced *XBP1* (*sXBP1*) mRNA (Fig. 1e and S3a Fig.) and ATF4 protein (Fig. 1f & g and S3b–f Fig.) respectively.

The expression of p.N617K induced increased levels of *sXBP1* mRNA indicating increased IRE1 activity. *ATF6*α knockdown further increased the level of *sXBP1* in the p.N617K expressing cells (Fig. 1e) but either decreased slightly, or had no effect on, the levels of *sXBP1* in cells expressing the other mutant forms of collagen X (S3a Fig.). It is noteworthy that in all cases, knockdown of *ATF6*β prevented the increase in *sXBP1* induced by the expression of the mutant forms of collagen X (Fig. 1e; S3a Fig.).

Increased levels of ATF4 protein are indicative of increased PERK signalling [29]. The expression of the p.N617K MCDS-causing collagen X construct resulted in a significant increase in ATF4 protein compared to the un-transfected controls (Fig. 1f & g). *ATF6*α knockdown further increased the levels of ATF4 whereas *ATF6*β knockdown had no effect (Fig. 1f & g). Representative western blots of ATF4 protein in cells expressing the different forms of mutant collagen are shown (Fig. 1g & S3 c–f Fig.) along with the levels of intracellular collagen X accumulation in the same cell extracts. There was a clear reciprocal relationship between the levels of collagen X and the levels of ATF4 in each transfected cell extract. When ATF4 was high, indicating increased PERK signalling and a decrease in general protein synthesis due to increased eIF2α phosphorylation, the level of intracellular collagen X was reduced and vice versa (Fig. 1g & S3c–f Fig.).

These latter results suggest that the loss of *ATF6*α causes an increase in ER stress level indicated by increased PERK signalling which accounts for both the increased ATF4 protein and the reduced synthesis of the mutant collagen X. The reduced level of collagen X accumulation following *ATF6*α knockdown was not caused by decreased *COL10A1* mRNA levels as these were unaffected by the knockdown of either isoform (S1g Fig.) and are unlikely to have been caused by increased secretion since the mutant proteins are largely retained in the ER in cell culture models [30]. The fact that that *ATF6*β knockdown prevented the MCDS-induced increase in *sXBP1* suggests that the UPR is more effective in the absence of ATF6β, possibly due to its previously reported capacity to attenuate the activity of ATF6α [25,26].

Hence, the *sXBP1* and ATF4 results suggest that ATF6α operates in a more efficient manner in terms of managing increased levels of ER stress in the absence of ATF6β. On the basis of the cell culture experiments described above, we hypothesized that ablation of *Atf6*α in the mouse MCDS model (*Col10a1*p.N617K) would cause a more severe-, and ablation of *Atf6*β a milder-, phenotype respectively. We therefore crossed the MCDS and *Atf6*α^−^^/^^−^ or *Atf6*β^−^^/^^−^ lines and phenotyped the resulting mice.

### *ATF6*α ablation increases disease severity in the ER stress-related dwarfism MCDS

In the first instance, we made a detailed study of the growth and growth plate histology of the *Atf6*α^−^^/^^−^ mouse to ensure there were no subtle skeletal phenotypes that could have been missed on initial investigation [18,19]. The growth plates of *Atf6*α^−^^/^^−^ mice were indistinguishable from the wild type equivalents based on appearance and histomorphometry (S4a & b Fig.). The body weights and bone lengths of wild type and knockout mice at 3 weeks of age were also indistinguishable (S4c & d Fig.).

The body weights of the MCDS mice (homozygous for the *Col10a1*p.N617K mutation) with ablated *Atf6*α (hereafter referred to as *Atf6*α^−^^/^^−^/MCDS) were slightly, albeit mostly not significantly, lower than their *Atf6*α^+/+^ counterparts (*Atf6*α^+/+^/MCDS) at 3 and 6 weeks of age (Table 1). Skeletal analysis at 3 and 6 weeks of age revealed significant reductions in the endochondral bone lengths of both male and female *Atf6*α^−^^/^^−^/MCDS mice (Table 1). These differences were still apparent at 9 weeks of age but had dropped below statistical significance in the male tibias (Table 1). The inner canthal distance (ICD) is determined by intramembranous bone growth and was not altered by either MCDS [7] or by *Atf6*α ablation (Table 1) demonstrating that the reduced bone lengths in endochondral bones (e.g. tibia and femur) are related to a growth plate defect and do not result from a more generalized effect on bone growth.

**Table 1:** Effects of *Atf6*α ablation on the skeletal development and body weights of MCDS mice over a 9 week period.

	Body measurements	Genotypes	3 weeks	6 weeks	9 weeks
Male	Weight (g)	*Atf6*α^+/+^/MCDS	9.7 ± 0.3 (15)	19.3 ± 0.5 (13)	22.3 ± 0.1 (5)
		*Atf6*α^−/−^/MCDS	9.0 ± 0.5 (8)	18.8 ± 0.6 (11)	22.6 ± 0.8 (5)
	Femur length (mm)	*Atf6*α^+/+^/MCDS	8.8 ± 0.1 (8)	10.8 ± 0.1 (9)	11.8 ± 0.1 (6)
		*Atf6*α^−/−^/MCDS	8.4 ± 0.1 (6)*	9.9 ± 0.2 (9)***	11.1 ± 0.2 (5)**
	Tibia Length (mm)	*Atf6*α^+/+^/MCDS	10.4 ± 0.1 (8)	12.0 ± 0.1 (9)	12.5 ± 0.1 (6)
		*Atf6*α^−/−^/MCDS	9.7 ± 0.1 (6)***	11.1 ± 0.3 (9)***	12.0 ± 0.1 (5)
	ICD (mm)	*Atf6*α^+/+^/MCDS	5.0 ± 0.01 (8)	5.1 ± 0.01 (9)	5.2 ± 0.1 (6)
		*Atf6*α^−/−^/MCDS	5.0 ± 0.04 (6)	5.1 ± 0.01 (9)	5.1 ± 0.2 (5)
Female	Weight (g)	*Atf6*α^+/+^/MCDS	9.0 ± 0.2 (18)	16.2 ± 0.3 (13)	17.3 ± 0.2 (8)
		*Atf6*α^−/−^/MCDS	8.2 ± 0.3 (24)	15.4 ± 0.6 (7)	17.6 ± 0.3 (5)
	Femur length (mm)	*Atf6*α^+/+^/MCDS	8.6 ± 0.1 (15)	10.4 ± 0.1 (13)	11.2 ± 0.1 (9)
		*Atf6*α^−/−^/MCDS	8.1 ± 0.1 (8)**	9.3 ± 0.1 (7)***	10.8 ± 0.2 (5)**
	Tibia Length (mm)	*Atf6*α^+/+^/MCDS	10.1 ± 0.1 (15)	11.6 ± 0.2 (13)	12.3 ± 0.1 (9)
		*Atf6*α^−/−^/MCDS	9.4 ± 0.2 (8)****	9.9 ± 0.2 (7)***	11.5 ± 0.3 (5)****
	ICD (mm)	*Atf6*α^+/+^/MCDS	5.0 ± 0.03 (15)	5.1 ± 0.01 (13)	5.2 ± 0.1 (9)
		*Atf6*α^−/−^/MCDS	5.0 ± 0.01 (8)	5.1 ± 0.01 (7)	5.2 ± 0.1 (5)

Mean ± SEM (n): *p < 0.05, **p < 0.01, ***p < 0.001, and ****p < 0.0001 when compared to *Atf6*α^+/+^/MCDS. All statistical analysis by ANOVA. (ICD = Inner canthal distance).

### *Atf6*α ablation increases MCDS-associated expansion of the growth plate hypertrophic zone (HZ)

**Fig. 2:**
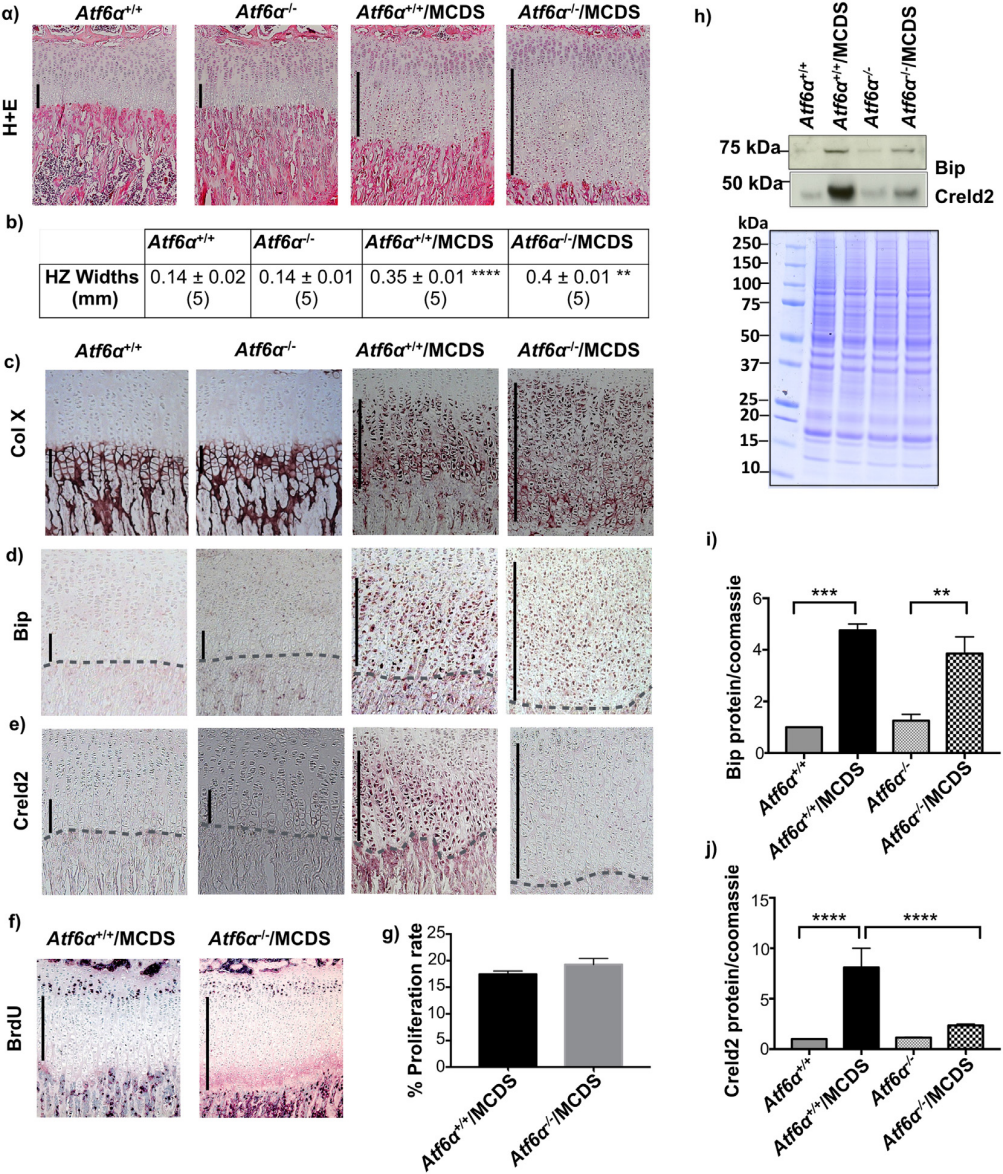
Effects of *Atf6*α ablation on the growth plate pathology associated with MCDS. (a) H & E staining of tibial growth plat. (b) Widths of hypertrophic zones at three weeks of age. Mean ± SEM (N) (**p < 0.01 compared to *Atf6*α^+/+^/MCDS, ****p < 0.001 compared to *Atf6*α^+/+^). Immunohistochemistry for (c) collagen X, (d) Bip, and (e) Creld2 in three week old mice with specified genotypes. The vertical black lines delineate the hypertrophic zones. (f) Images represent immunohistochemistry for BrdU performed on tibial growth plate sections of three week old MCDS mice that were either wild type of knockout for *Atf6*α. (g) Percentage of BrdU labelled nuclei (black stained) calculated against the total number of cells in the proliferative zone. Mean ± SEM (n = 5). (h) A representative western blots of rib growth plate extracts at three weeks of age for Bip and Creld2. Coomassie blue stained gel was used as loading control. (i & j) Quantification of Bip and Creld2 from three independent analysis. All statistical analysis by ANOVA (**p < 0.01, ***p < 0.001 and ****p < 0.0001

As has been noted previously, the growth plate HZs of *Atf6*α^+/+^/MCDS mice were approx. 2.5-fold wider than their non-MCDS wild type equivalents at 3 weeks of age. Nevertheless, the ablation of *Atf6*α resulted in a further 14% expansion of the HZ of MCDS mice (Fig. 2a & b; p < 0.01) which is indicative of more severe ER stress in the *Atf6*α^−^^/^^−^/MCDS mouse since the degree of HZ expansion in MCDS directly and positively correlates with the disease severity [7,8,28].

Although the HZ zone is wider in the *Atf6*α^−^^/^^−^ compared to *Atf6*α^+/+^/MCDS mouse, the pattern of collagen X retention was comparable (Fig. 2c). In contrast, the immunohistochemical staining for Bip in the MCDS HZ was more intense in the *Atf6*α^+/+^ compared to *Atf6*α^−^^/^^−^ growth plate (Fig. 2d). Quantitation of Bip in rib growth plate extracts by western blotting did not reveal a significant difference in the levels between the Aft6 sufficient and deficient MCDS mice (Fig. 2h & i), whereas mRNA levels, assessed by qPCR were higher in the *Atf6*α^+/+^ MCDS mice (S6a Fig.). The upregulation of HZ Creld2 apparent by immunolocalisation in MCDS mice was markedly suppressed by *Atf6*α ablation (Fig. 2e) and similar results were obtained by western blotting (Fig. 2h & j) and qPCR (S6b Fig.) of rib growth plate extracts. BrdU labelling revealed no differences in growth plate chondrocyte proliferation between *Atf6*α^+/+^/MCDS and *Atf6*α^−^^/^^−^/MCDS mice (Fig. 2f & g).

### *Atf6*α ablation decreases growth plate histomorphometric markers of bone growth in MCDS mice

**Fig. 3:**
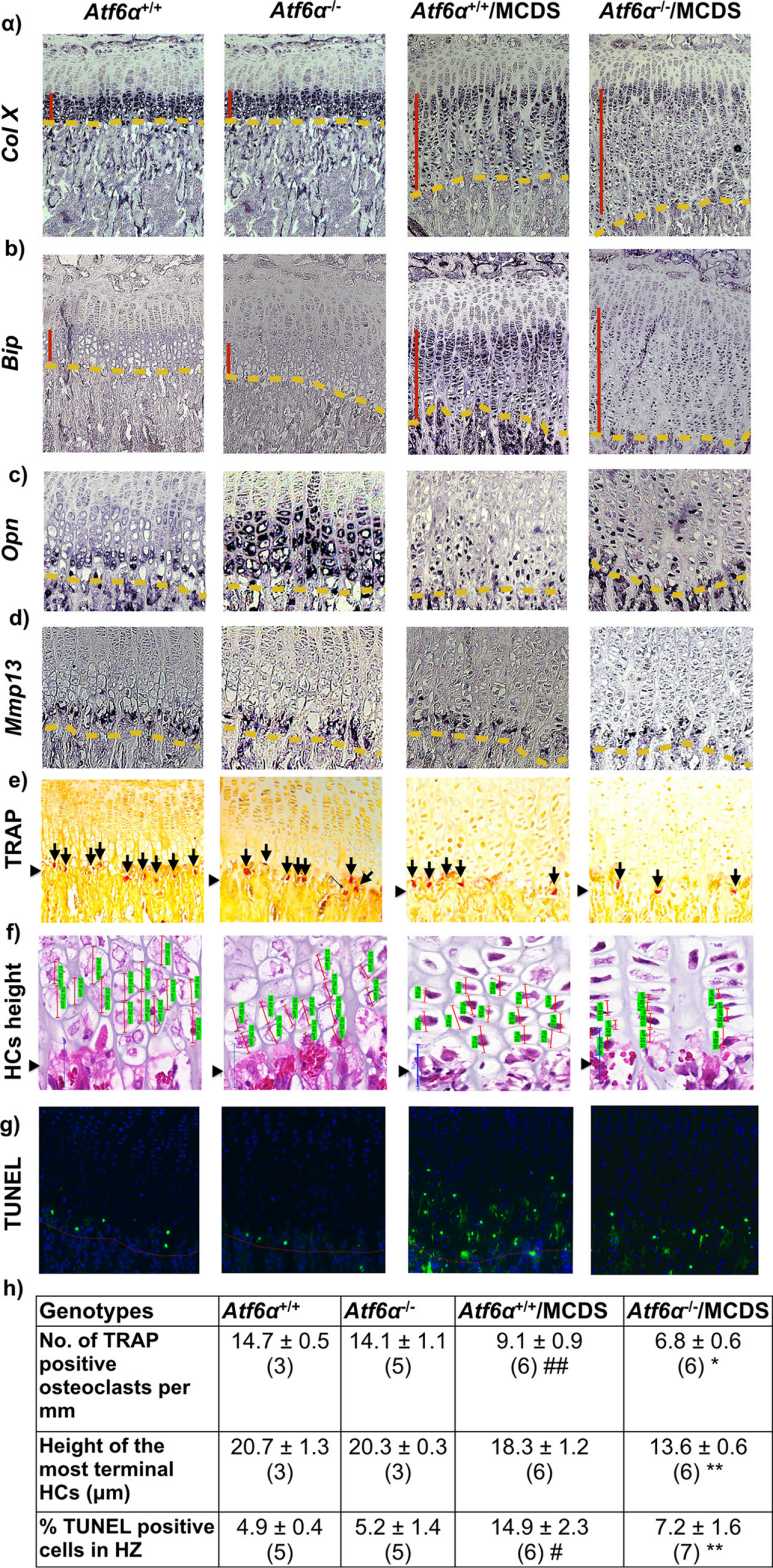
Effects of *Atf6*α ablation on chondrocytes differentiation. Tibial growth plates from three week old mice with specified genotypes were analysed for the expression of mRNAs encoding (a) *Col10a1*, (b) *Bip*, (C) *Osteopontin*, and (d) *Mmp13*. The presence of transcript is indicated by the dark blue staining. The hypertrophic zone is indicated by the vertical red line and the vascular invasion front by the yellow dashes. (e) TRAP staining for osteoclasts (arrows) at the vascular invasion front (arrow head). The number of osteoclast per mm of vif is represented in the table h. (f) Snapshot of measurement of height of the most terminal hypertrophy chondrocytes (HCs) for each specific genotype. HCs are indicated by the red vertical lines and their corresponding measurements are highlighted in green. (arrow head = vif). Table (h) represents the average heights of the most terminal HCs. (g) TUNEL assay on tibial growth plate sections of three week old animals with specific genotypes. (Green stained cells = apoptotic cells, blue-stained cells = DAPI, red dotted lines = vascular invasion front). Table (h) shows the number of apoptotic HCs as a percentage of the total number of chondrocytes within HZ. (h) Mean ± SEM (N) (*p < 0.05 and **p < 0.01 compared to *Atf6*α^+/+^/MCDS, #p < 0.01 and # #p < 0.001 compared to *ATF6*α^+/+^). All statistical analysis by ANOVA. (For interpretation of the references to colour in this figure legend, the reader is referred to the web version of this article.)

Whereas the expression of *Col10a1* mRNA was apparent as a neat band in all hypertrophic chondrocytes apart from the most terminal in the non-MCDS wild type controls (*Atf6*α^+/+^ & *Atf6*α^−^^/^^−^), the pattern of expression was disrupted and more sporadic in MCDS. *Atf6*α ablation had little influence in the MCDS mouse pattern in comparison with the *Atf6*α^+/+^/MCDS control (Fig. 3a). *Bip* mRNA was clearly highly upregulated in the expanded growth plate of the *Atf6*α^+/+^/MCDS mouse whereas the induction of *Bip* was noticeably less intense in the *Atf6*α^−^^/^^−^/MCDS growth plate HZ (Fig. 3b; see also S6a Fig.). Osteopontin (*Opn*) mRNA expression is normally limited to the most terminally differentiated hypertrophic chondrocytes adjacent to the vascular invasion front (see Fig. 3c - *Atf6*α^+/+^ panel). Surprisingly, *Atf6*α^−^^/^^−^ (non-MCDS) mice expressed *Opn* mRNA in hypertrophic chondrocytes throughout the HZ zone (Fig. 3c). In MCDS mice expressing *Atf6*α, terminal differentiation becomes cell autonomous and *Opn* becomes expressed sporadically by hypertrophic chondrocytes in the lower half of the HZ as cells complete their differentiation (Fig. 3c) [7,8]. In MCDS (unlike on a wild-type collagen X background), *Atf6*α^−^^/^^−^ did not affect *Opn* expression in comparison to the *Atf6*α^+/+^/MCDS control (Fig. 3c). Similarly to *Opn*, *Mmp13* mRNA was also restricted to the most terminal hypertrophic chondrocytes in wild type mice but was expressed sporadically by individual hypertrophic chondrocytes in the HZ of *Atf6*α^+/+^/MCDS mice (Fig. 3d) [7,8]. Again *Atf6*α ablation did not affect the expression of *Mmp13* in *Atf6*α^−^^/^^−^/MCDS mice (Fig. 3d).

**Fig. 4:**
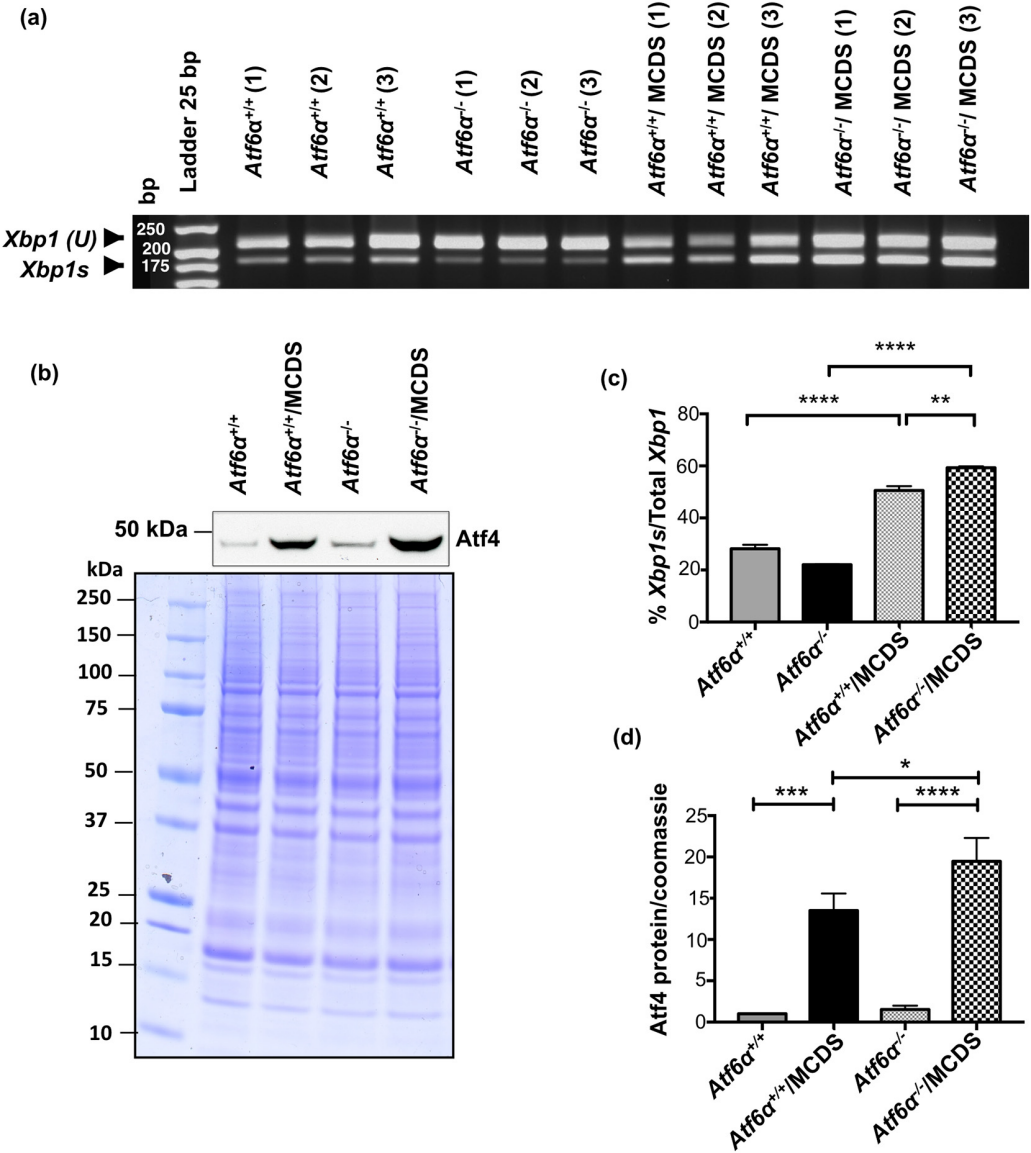
Effects of *Atf6*α ablation on the activities of PERK and IRE1 signalling pathways. (a) Total RNA from three pooled ribs growth plate extracts of 21-day old mice were extracted and subjected to qPCR with XBP1 primers. Gel shows three independent samples for each genotype. Upper bands on the gel indicates *Xbp1*(*U*) with the size of 205 bp and lower band is Xbp1s (size = 179 bp). (b) A typical western blots of rib growth plate extracts at three weeks of age for Atf4. Coomassie blue stained gel was used as loading control. (C) The average rate of Xbp1 splicing from five independent samples for each genotype. (d) Quantification of Atf4 from five independent analyses (*p < 0.05, **p < 0.01, ***p < 0.001, ****p < 0.0001).

The expansion of growth plate width in MCDS is due to decreased osteoclast recruitment to the vascular invasion front resulting in a decreased rate of hypertrophic cartilage erosion [7]. We therefore quantified the effects of *Atf6*α ablation on osteoclast recruitment to the vascular invasion front by staining the osteoclasts with tartrate resistant alkaline phosphatase (TRAP). *Atf6*α ablation in mice wild type for *Col10a1* had no effect on osteoclast numbers at the vascular invasion front (Fig. 3e & h). As described previously, MCDS (*Atf6*α^+/+^/MCDS) reduced osteoclast recruitment approx. 30% and notably, *Atf6*α ablation caused a further significant 20% fall in TRAP-stained cells at the vascular invasion front in *Atf6*α^−^^/^^−^/MCDS mice (Fig. 3e & h). This reduction in osteoclasts accounts for the further expansion in HZ width seen in the MCDS mice lacking *Atf6*α. Longitudinal bone growth is in large part determined by the size or “height” that the hypertrophic chondrocytes achieve during terminal differentiation [31]. The heights achieved by the hypertrophic chondrocytes in the *Atf6*α^−^^/^^−^/MCDS mice were significantly less than equivalent cell heights achieved in the *Atf6*α^+/+^/MCDS HZ (Fig. 3f & h) accounting for the reduced bone lengths seen in the MCDS mice that lacked Atf6 (Table 1). In wild type mice, apoptosis is usually restricted to the terminal hypertrophic chondrocytes in the vicinity of the vascular invasion front (*Atf6*α^+/+^ − Fig. 3g). Atf6α ablation on a wild type collagen X background did not alter this distribution (*Atf6*α^−^^/^^−^ − Fig. 3g). As previously noted in a recent study [9] MCDS caused an increase in Tunnel-positive apoptotic cells distributed through the lower half of the HZ (*Atf6*α^+/+^/MCDS - Fig. 3g & h). *Atf6*α ablation significantly reduced the numbers of apoptotic chondrocytes distributed throughout the lower half of the HZ (*Atf6*α^−^^/^^−^/MCDS – Fig. 3g & h) presumably due reduced expression of Atf6α-dependent *Chop* (see Fig. 1b in vitro and S6c Fig. in vivo). The reduction in *Chop*, which correlates with apoptosis rates in vivo is intriguing given that both in vitro (Fig. 1f) and in vivo (Fig. 4b & d) ATF4 levels were increased in MCDS cells/tissues lacking *ATF6*α. These data strongly suggest that the induction of CHOP is more dependent upon ATF6α than ATF4.

### *Atf6*α ablation in MCDS mice increases growth plate ER stress based on measures of signalling through the Ire1 and Perk pathways

The levels of *Xbp1* mRNA splicing (*Xpb1s*) were assessed in rib growth plate extracts as a measure of signalling through the Ire1 pathway. MCDS caused an increase in the proportion of *Xpb1s* present in samples (*Atf6*α^+/+^/MCDS) and the levels of splicing were further increased in the *Atf6*α^−^^/^^−^/MCDS mice (Fig. 4a & c). Signalling through the Perk pathway was assessed by measuring the levels of Atf4 protein in rib extracts by western blotting. Similar to the *Xpb1s*, MCDS caused an increase in the level of Atf4 protein (*Atf6*α^+/+^/MCDS – Fig. 4b & d) with *Atf6*α ablation inducing a further significant increase (*Atf6*α^−^^/^^−^/MCDS mice - Fig. 4b & d). Therefore, *Atf6*α ablation resulted in increased signalling through both the Ire1 and Perk pathways in MCDS mice.

In summary, *Atf6*α ablation resulted in a more severe phenotype in MCDS mice based on decreased rates of bone growth (Table 1), histopathology, histomorphometry and histochemistry (Fig. 2), decreased markers of hypertrophic chondrocyte differentiation (Fig. 3) and biochemical evidence for increased signalling flux through the Perk and Ire1 pathways (Fig. 4). Paradoxically, the *Atf6*α^−^^/^^−^/MCDS mice exhibited a reduced level of apoptosis in the HZ compared to the Atf6-sufficient MCDS mice suggesting that hypertrophic chondrocyte apoptosis per se has no direct influence on the disease severity in this disease. We next tested the second part of our hypothesis, namely that *Atf6*β ablation would decrease the disease severity in MCDS.

### Effects of *Atf6*β ablation on normal mouse skeletal growth

**Fig. 5:**
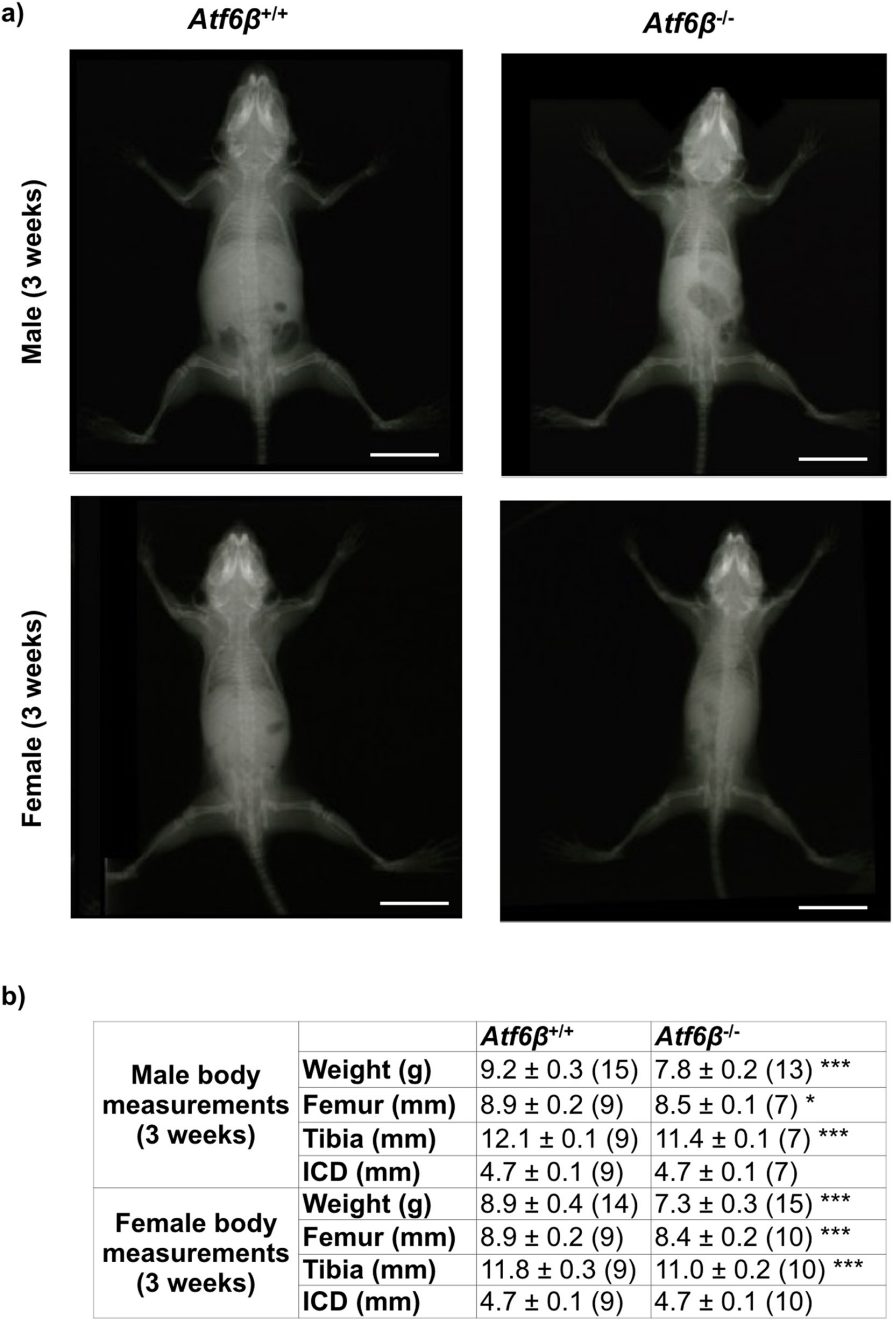
Effects of *Atf6*β ablation on the skeletal development and body weight of three week old mice. (a) A representative X-ray radiograph image for three-week old male mice (top row) and female mice (bottom row) with the specified genotypes. White scale bar = 100 μm (b) Mean ± SEM (N). *p < 0.05 and ***p < 0.001 when compared to *Atf6*β^+/+^mice. All statistical analysis by ANOVA.

**Fig. 6:**
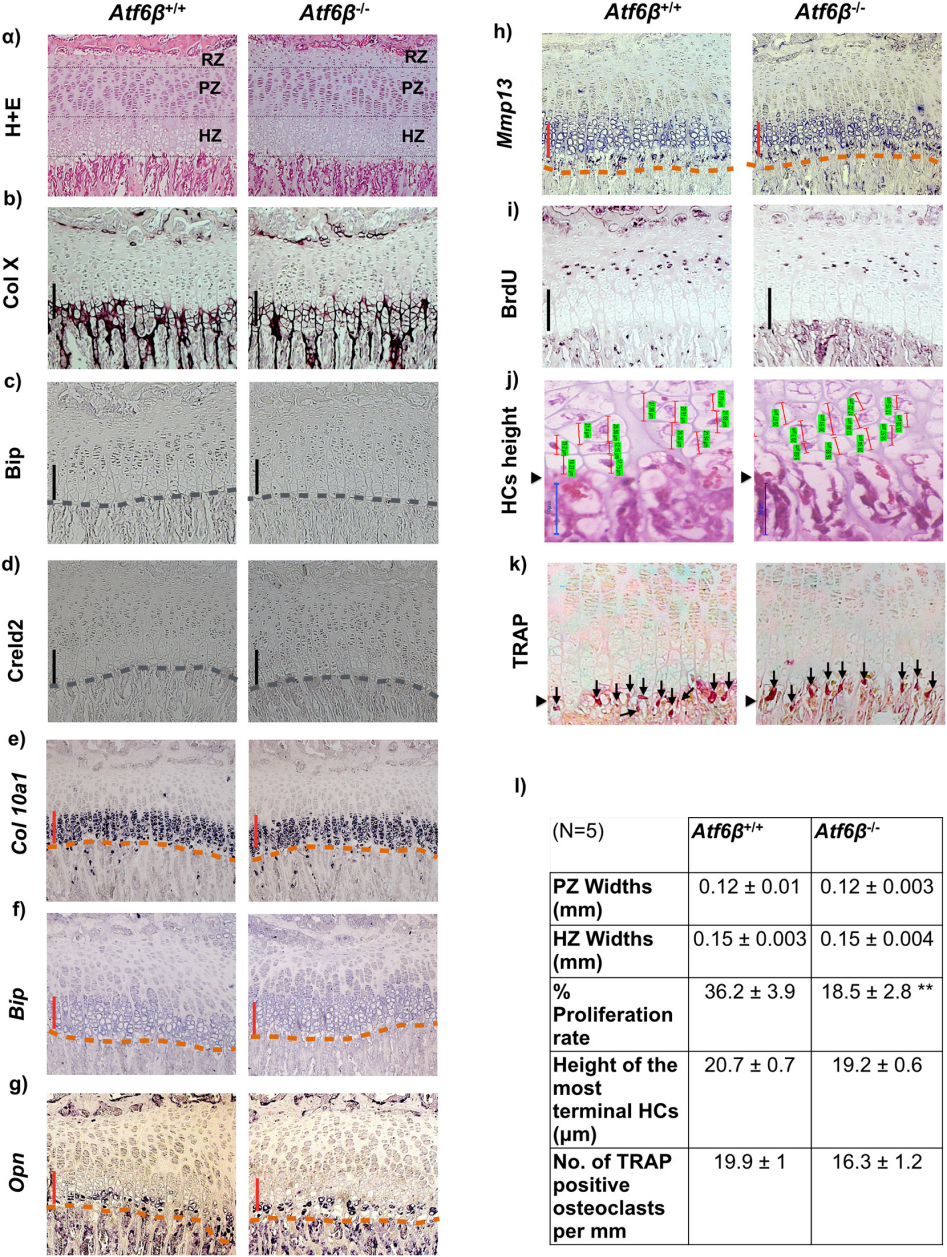
Analysis of growth plate in three week old *Atf6*β^−/−^ mice. (a) H & E staining of tibial growth plate and immunohistochemistry for (b) collagen X, (c) Bip, and (d) Creld2. The vertical black lines delineate the hypertrophic zones. Grey dashed lines indicated vascular invasion front. In situ hybridisation for (e) *Col10a1*, (f) *Bip* (g) *osteopontin*, and (h) *Mmp13* mRNAs. The presence of transcript is indicated by the dark blue staining. HZ is shown by the vertical red lines. (i) 5-bromo-29-deoxyuridine (BrdU) labelling of proliferative cells in the growth plate. Positive cells stained black. (j) Snapshots of height measurement in the most terminal hypertrophic chondrocytes (HCs). Hypertrophic chondrocytes are indicated by red vertical lines and their corresponding measurements are highlighted in green. Scale bar = 50 μm. (k) TRAP staining for osteoclasts (arrows). Closed arrow head = vascular invasion front. (l) Mean ± SEM (N = 5). **p < 0.01 as determined by ANOVA.

To ensure there were no subtle skeletal phenotypes caused by *Atf6*β ablation that may have been missed during the initial phenotyping, we measured body weights, and long bone lengths from x-rays of 3 week old mice. Surprisingly, we found that for *Atf6*β knockouts, both male and female mice were slightly, but significantly lighter and their tibias and femurs were also slightly shorter than their wild type controls (Fig. 5). The inner canthal distances of the knockouts and controls however were identical showing that the skeletal effects of *Atf6*β ablation were on endochondral but not intramembranous bone formation. We next conducted a growth plate examination of wild type (*Atf6*β^+/+^) and knockout (*Atf6*β^−^^/^^−^) mice. The histological appearance, histomorphometry of the different zones, immunolocalisation of collagen X, Bip and Creld2 and gene expression of *Col10a1*, *Bip*, *Creld2*, *Opn* and *Mmp13*, were indistinguishable between the wild type and *Atf6*β^−^^/^^−^ mice (Fig. 6a–h and l). However, BrdU labeling revealed a significant decrease in the proliferation rate of growth plate chondrocytes in the *Atf6*β^−^^/^^−^ mice (Fig. 6i & l) whereas the height of the terminal hypertrophic chondrocytes (Fig. 6j & l) and the numbers of TRAP-positive osteoclasts recruited to the vascular invasion front (Fig. 6k & l) did not significantly differ between the genotypes. Accordingly, we concluded that the reduced bone growth in the *Atf6*β knockout mouse was caused by a proliferative zone defect.

Clearly, this would confound interpretation of growth and skeletal measurements of *Atf6*β^−^^/^^−^/MCDS mice since any differences from the *Atf6*β^+/+^/MCDS would be the compound result of both a proliferative and a hypertrophic zone defect. Nevertheless, direct comparisons of the severity of the growth plate hypertrophic zone phenotypes of the 2 MCDS lines would still provide insights into some aspects of the ER stress being experienced in the presence and absence of Atf6β.

### *Atf6*β^−^^/^^−^/MCDS and *Atf6*β^+/+^/MCDS mouse body weight and bone growth data

*Atf6*β^−^^/^^−^/MCDS mice were lighter and had shorter long bones than their *Atf6*β^+/+^/MCDS controls. As discussed above, since the *Atf6*β^−^^/^^−^/MCDS mice have a proliferative zone defect that was not present in the *Atf6*β^+/+^/MCDS mice, interpretation of the data is confounded. Therefore, the data set is presented in S5 Fig. for the sake of completeness but is not discussed further here.

### Ablation of *Atf6*β decreased the severity of the growth plate HZ pathology in MCDS mice

**Fig. 7:**
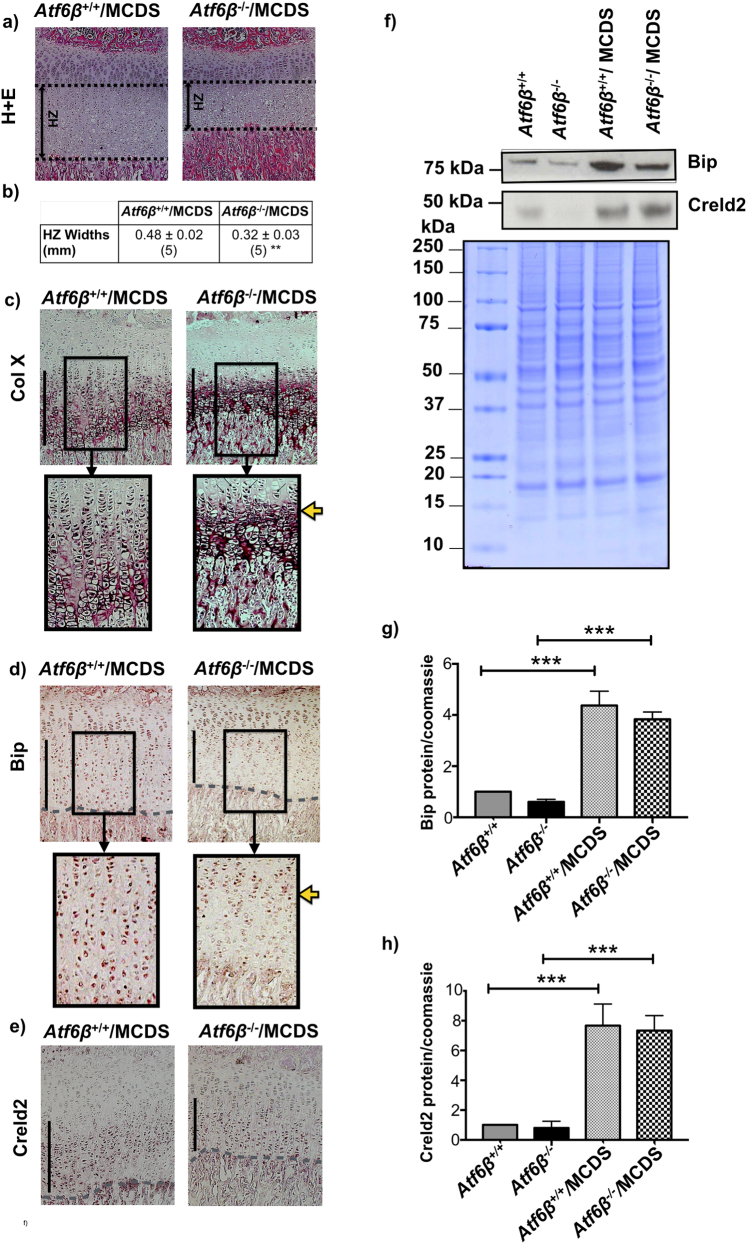
Effects of *Atf6*β ablation on the growth plate pathology associated with MCDS. (a) H & E staining of tibial growth plate in three week old MCDS mice that were either wild type or knockout for *Atf6*β. (b) Widths of hypertrophic zones at three weeks of age. Mean ± SEM (N) (**p < 0.01 as determined by ANOVA). Immunohistochemistry for (c) collagen X, (d) Bip, and (e) Creld2. The vertical black lines delineate the hypertrophic zones The black boxed photomicrographs represent an expanded view of the indicated areas within hypertrophic zones in the sections from specified genotypes. The intracellular accumulation of collagen X (black arrows) is only apparent in the upper hypertrophic zone in *Atf6*β^−/−^/MCDS sample. The yellow horizontal arrow in the expanded view of *Atf6*β^−/−^/MCDS sample indicates the transition region in which; (c) intracellular accumulation of collagen X protein is resolved and collagen X protein is secreted (d) Bip is down-regulated. (f) A representative western blots of rib growth plate extracts at three weeks of age for BiP and CRELD2. Coomassie blue stained gel was used as loading control. (g & h) Quantification of Bip and Creld2 from three independent analysis (***p < 0.001). (For interpretation of the references to colour in this figure legend, the reader is referred to the web version of this article.)

Histological examination revealed that the MCDS-induced expansion in the width of the HZ was significantly reduced in the *Atf6*β^−^^/^^−^/MCDS mice compared to their *Atf6*β^+/+^/MCDS controls (Fig. 7a & b). The intracellular retention of mutant collagen X in *Atf6*β^−^^/^^−^/MCDS mice was limited to the upper part of the HZ but extended much further in the *Atf6*β^+/+^/MCDS growth plate. The apparent increased intensity of immune-stain in the mice lacking *Atf6*β may indicate more efficient secretion of the mutant collagen X into the ECM (Fig. 7c). Immunostaining for Bip was restricted and limited to the upper region of the HZ in *Atf6*β^−^^/^^−^/MCDS mice (Fig. 7d) whereas Creld2 staining was similar in *Atf6*β^+/+^/MCDS and *Atf6*β^−^^/^^−^/MCDS mice extending throughout the HZ (Fig. 7e). Western blotting and qPCR of rib growth plate extracts revealed that both Bip and Creld2 were significantly increased in the MCDS mice but the level of Bip and Creld2 were not significantly altered by *Atf6*β ablation in MCDS mice (Fig. 7f–h; S6d & e Fig.).

### *Atf6*β ablation ameliorated HZ markers of deficient differentiation and reduced bone growth in MCDS mice

**Fig. 8:**
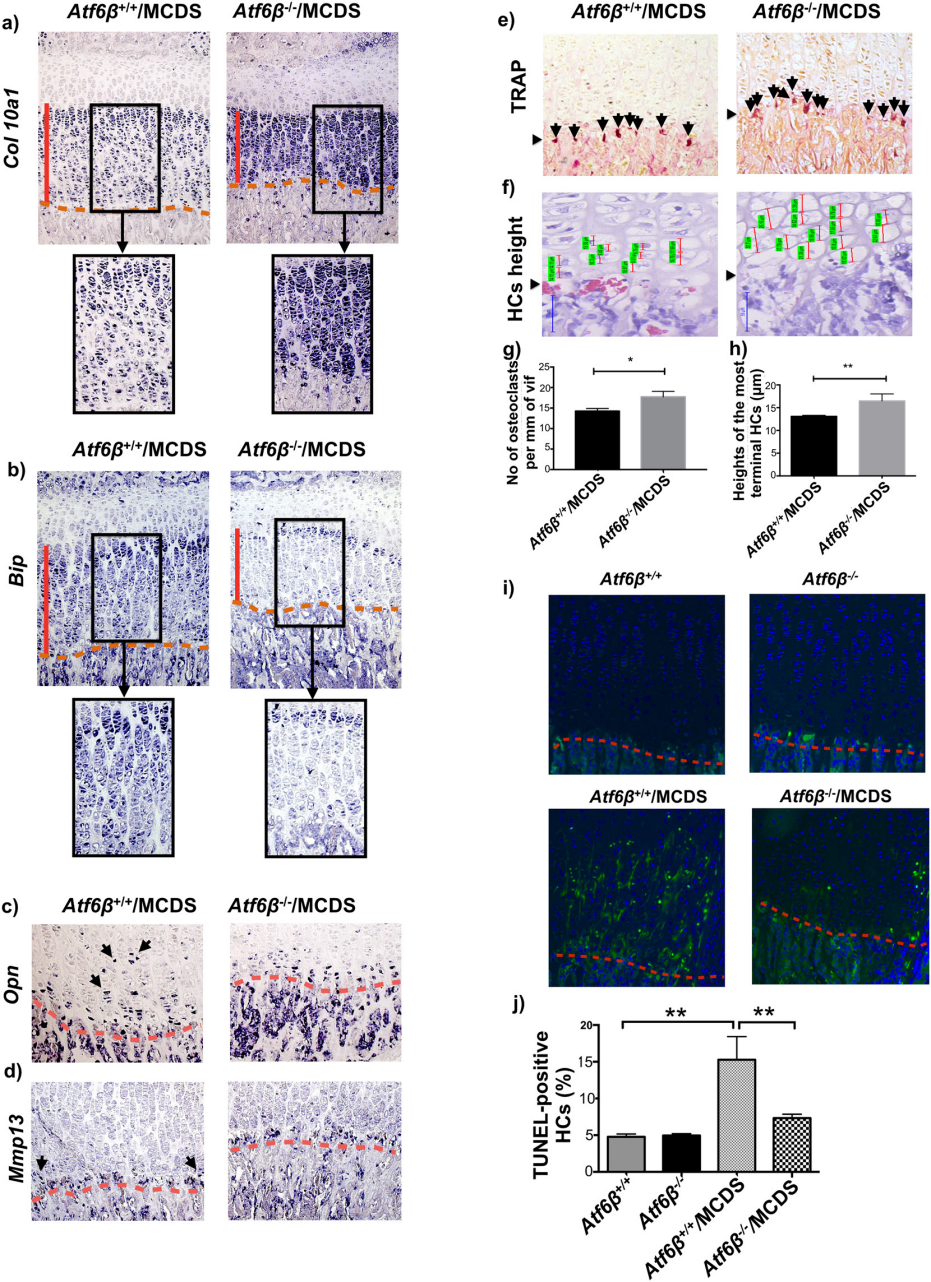
Effects of *Atf6*β ablation on chondrocytes differentiation. In situ hybridisation for (a) *collagen X*, (b) *Bip*, (C) *Osteopontin* (black arrows), and (d) *Mmp13* (black arrows) in tibial growth plate sections from three week old MCDS mice with specified genotypes The presence of transcript is indicated by the dark blue staining. The hypertrophic zone is indicated by the vertical red line and the vascular invasion front by the red dashes. The black boxed photomicrographs represent an expanded view of the indicated areas within hypertrophic zones in the sections from specified genotypes. (e) TRAP staining for osteoclasts (arrows) at the vascular invasion front (arrow head). (f) Snapshot of measurement of height of the most terminal hypertrophy chondrocytes (HCs) for each specific genotype. HCs are indicated by the red vertical lines and their corresponding measurements are highlighted in green. (arrow head = vif). (g) The number of osteoclast per mm of vif. Mean ± SEM (5). *p < 0.05. (h) The average heights of the most terminal HCs. Mean ± SEM (5). **p < 0.01 (i) TUNEL assay on tibial growth plate sections of three week old mice with specific genotypes. (Green stained cells = apoptotic cells, blue-stained cells = DAPI, red dotted lines = vascular invasion front). Table (j) The number of apoptotic HCs as a percentage of the total number of chondrocytes within HZ. Mean ± SEM (*N* ≥ 3). **p < 0.01. (k) qPCR for Chop on ribs growth plate extracts of three week old mice. Mean ± SEM (3). ****p < 0.0001 as determined by one way ANOVA. (For interpretation of the references to colour in this figure legend, the reader is referred to the web version of this article.)

*Col10a1* mRNA expression was more intense and continuous throughout the HZ of the *Atf6*β^−^^/^^−^/MCDS mice compared to the *Atf6*β^+/+^/MCDS controls that displayed the typical interrupted expression pattern usually associated with MCDS (Fig. 8a). *Bip* expression was focused to the prehypertrophic and very first hypertrophic chondrocytes and then down-regulated in the remainder of the HZ apart from a few sporadic cells in animals lacking *Atf6*β (*Atf6*β^−^^/^^−^/MCDS - Fig. 8b). In contrast, the sporadic expression of *Bip* mRNA was far stronger in the HZ of the control MCDS mice (*Atf6*β^+/+^/MCDS – Fig. 8b).

The expression of *Opn* mRNA was more focused to the terminal hypertrophic chondrocytes adjacent to the vascular invasion front in MCDS lacking *Atf6*β^−^^/^^−^ (Fig. 8c) whereas Mmp13 expression was similar in the *Atf6*β^+/+^/MCDS and *Atf6*β^−^^/^^−^/MCDS mice (Fig. 8d). Where the *Atf6*β^−^^/^^−^/MCDS differ from their *Atf6*β-sufficient equivalents, the changes in expression together with the reduced width of the HZ are indicative of improved hypertrophic chondrocyte differentiation more reminiscent of that seen in wild type (non MCDS) mice. In addition, quantitation of osteoclast recruitment to the vascular invasion front by TRAP staining (Fig. 8e and g) and measurement of hypertrophic cell “height” (Fig. 8f & h) also revealed that *Atf6*β ablation at least partially ameliorated the MCDS-induced decreases in these histological indicators of bone growth, again indicative of improved hypertrophic chondrocyte differentiation.

*Atf6*β ablation in wild type (non MCDS) mice did not affect apoptosis as judged by TUNEL staining which was restricted to hypertrophic chondrocytes at the vascular invasion front (Fig. 8i). The increased apoptosis in the hypertrophic zone of MCDS mice replete with both forms of Atf6 (*Atf6*β^+/+^/MCDS – Fig. 8i; *Atf6*α^+/+^/MCDS – Fig. 3g) was significantly reduced in *Atf6*β^−^^/^^−^/MCDS mice (Fig. 8i and j). This was accompanied by a significant fall in the level of *Chop* mRNA in the *Atf6*β^−^^/^^−^/MCDS compared to *Atf6*β^+/+^/MCDS mouse growth (S6f Fig.).

### *Atf6*β ablation in MCDS mice decreases growth plate ER stress based on measures of signalling through the Ire1 and Perk pathways

**Fig. 9:**
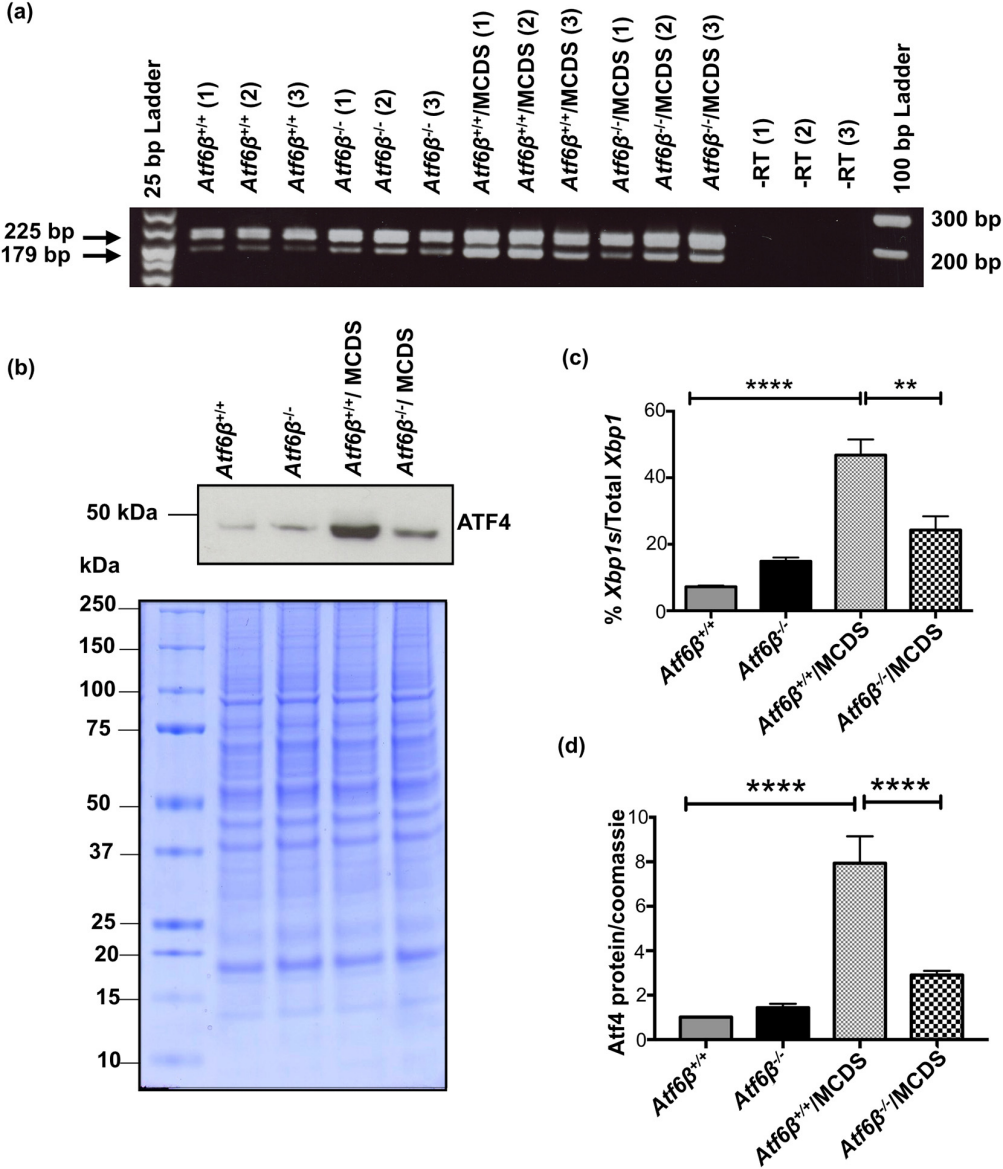
Effects of *Atf6*β ablation on the activities of PERK and IRE1 signalling pathways. (a) Total RNA from three pooled ribs growth plate extracts of 21-day old mice were extracted and subjected to qPCR with XBP1 primers. Three independent samples for each genotype are shown. Upper bands on the gel indicates *Xbp1*(U) with the size of 205 bp and lower band is *Xbp1s* (size = 179 bp). (−RT = minus reverse transcriptase control). (b) A typical western blots of rib growth plate extracts at three weeks of age for Atf4. Coomassie blue stained gel was used as loading control. (c) The average rate of Xbp1 splicing from three independent samples for each genotype. (d) Quantification of Atf4 from three independent experiments (**p < 0.01, ****p < 0.0001).

As expected, signalling through the Ire1 pathway, based on *sXbp1* mRNA levels, was significantly increased in the growth plate of control *Atf6*β^+/+^/MCDS mice. The ablation of *Atf6*β significantly reduced the MCDS-associated increased splicing of *Xbp1* (Fig. 9a & c). Similarly, signalling through the Perk pathway, assessed by measuring the level of Atf4 protein in growth plate extracts, was increased, as expected, in the control *Atf6*β^+/+^/MCDS mice but in comparison significantly decreased by *Atf6*β ablation (Fig. 9b & d). The reduced levels of signalling through both the Ire1 and Perk pathways and the reduced *Chop* (S6f Fig.) strongly suggest that the levels of ER stress are significantly reduced by *Atf6*β ablation and that this reduced ER stress is the mechanism responsible for the improved histological appearance of the growth plate in the *Atf6*β^−^^/^^−^/MCDS mice.

These findings from in vivo studies therefore support our original hypothesis that *Atf6*α ablation would be detrimental in terms of MCDS disease severity and that ablation of *Atf6*β would be beneficial.

## Discussion

While both ATF6α and -β undergo proteolytic cleavage and become activated following accumulation of unfolded/misfolded protein within the ER (ER stress), surprisingly only ATF6α has been implicated in the pathology of many ER stress-associated diseases and has been the main focus of most studies [[32], [33], [34], [35], [36], [37], [38], [39], [40], [41]]. Comparatively few studies have examined the function of ATF6β [11,18,20,[23], [24], [25], [26],^36^,^42^] and several of these have concluded that ATF6β either does not have any physiological significance or has a minor functional significance that is redundant with that of ATF6α [36,42]. The functional significance of ATF6β and its possible therapeutic potential has therefore been largely ignored in many ER stress-associated diseases, including MCDS.

### Shared and novel roles for ATF6α and ATF6β

Our analyses provide a direct and systematic comparison between ATF6α and -β in response to ER stress caused by a pathophysiologically relevant intracellular accumulation of misfolded mutant protein within the ER in the context of MCDS – a disease which is driven by increased ER stress and the resulting UPR [7,8,28]. The results provide new insight into the role of ATF6α and -β and interplay between them since, particularly in the in vivo part of the study, we could measure not only biochemical parameters of ER stress but concurrently assess the effects of Atf6 ablation on disease severity.

Firstly, it is clear from previous work that some of the functions of ATF6α and -β are not only vital for normal development but shared by both isoforms (redundant) since each isoform alone can support essentially normal development but ablation of both isoforms is embryonic lethal in mouse and fish [[18], [19], [20]]. Nevertheless, this does not preclude either or both isoforms of ATF6 acquiring exclusive responsibilities for ancestral roles (sub-functionalisation) or acquiring completely new roles (neo-functionalisation) although these latter roles cannot be essential since development is normal in the absence of either isoform.

Results presented in this study show that ATF6α is both induced by increased ER stress (S1 Fig.) and responsible for inducing the increased transcription of a range of ER stress-associated factors such as BiP, CHOP and ARMET whereas ATF6β is not (Fig. 1 and S2 Fig.) as has been firmly established by many others [11,18,19,25,26]. Another difference between ATF6α & β relates to their ability to induce CRELD2. The expression of the p.N617 K mutation caused a marked ATF6α-dependent increase in expression of CRELD2 in vitro (Fig. 1d) and in vivo (Fig. 2) that was unaffected by the presence of absence of ATF6β (Fig. 7). In addition, Atf6α appears responsible for suppressing the expression of *Opn* in all but terminally differentiated hypertrophic chondrocytes of wild type (but not MCDS) mice (Fig. 3c) whereas Atf6β plays an important role in controlling growth plate chondrocyte proliferation (Fig. 6i and l) although this latter effect may be dependent upon genetic background since the original Atf6β knockout mice were reported to be phenotypically normal [18].

The data presented here together with the evidence cited above in the introduction relating to liver steatosis in tunicamycin-challenged Atfα ko, but not Atf6β ko mice, and the exclusive role of Atf6β in regulating various genes, provides compelling evidence that each of the two ATF6 isoforms have acquired a number of exclusive roles since their divergence from a common ancestral ATF6. The biological function/significance of some of these new roles remain to be determined but they would appear to be wide-ranging.

### Competing roles of ATF6α and ATF6β in controlling ER stress?

The ER stress-related activities of ATF6α and β are intimately related but it is the ATF6α isoform that is a major transcriptional activator of ERSE genes and is a prerequisite for a robust UPR (Fig. 1 and S2 Fig.) and [11,18,20], while ATF6β appears to regulate the basal expression level of ATF6α-target genes. In addition, the transcriptional activity of ATF6α has been reported to be much greater than that of ATF6β [[25], [26], [27]]. In an attempt to gain insight into the overall effect of ATF6α & β knockdown on the level of ER stress experienced by cells expressing the different MCDS-causing mutant forms of collagen X, we measured the actual level of XBP1s mRNA (rather than the ratio of XBP1s: total XBP1 – see Materials and methods) as an indicator of IRE1 activity and ATF4 protein as a measure of PERK signalling. The knockdown of ATF6β completely prevented the mutant collagen X-induced increase in XBP1s indicating that ATF6α on its own effectively prevented the activation of the IRE1 pathway, an effect that could not be achieved in the presence of ATF6β either alone or in combination with ATF6α (Fig. 1e and S3a Fig.). In a similar and related fashion, knockdown of ATF6α (but not ATF6β) caused a marked increase in ATF4 protein and an accompanying decrease in collagen X synthesis (Figs. 1f & g, and S3b–f), indicators of increased PERK signalling, again suggesting a more pronounced role for ATF6α is controlling the level of ER stress. These data also indicate that the activity of ATF6α is dampened, rather than “anatagonised” as has been suggested previously [18] by the presence of ATF6β and the reason for this will be discussed later. Nevertheless, this effect of ATF6β on the activity of ATF6α led us to hypothesise that the MCDS phenotype in mice would be made more severe by Atf6α ablation and ameliorated by ablation of Atf6β.

*Atf6*α ablation in MCDS mice increased the overall levels of ER stress in the growth plate, as judged by IRE1 (*Xbp1* slicing) and PERK pathway (ATF4 protein) activities (Fig. 4) as was found in our earlier in vitro experiments (Fig. 1e–g and S3 Fig.). Concomitantly, disease severity in these mice was also increased based both on histological/histomorphometric analyses (Fig. 2), disruption to hypertrophic chondrocyte differentiation (Fig. 3) and decreased rates of long bone growth (Table 1). In contrast, *Atf6*β ablation in MCDS mice decreased overall levels of growth plate ER stress (Fig. 9) and decreased disease severity as judged by histological/histomorphometric analyses (Fig. 7) and by the improved hypertrophic differentiation apparent in the *Atf6*β-ablated mice (Fig. 8).

Accordingly, both our in vivo and in vitro data provide evidence that ATF6α most efficiently responds to and ameliorates increases in ER stress in the absence of ATF6β and that, in turn, a mix of ATF6α & β is the next most effective combination with ATF6β alone being least effective but why should this be? The two ATF6 isoforms need to dimerise to from an active transcription factor and they can form homodimers or heterodimers with each other or other factors such as XBP1s [17,26,43]. As described previously, the transcriptional activity of the ATF6α homodimer is far greater than that of ATF6β [18,20,[25], [26], [27]]. Therefore, the ATF6α/β heterodimer is likely to have an intermediate transcriptional activity assuming both isoforms are expressed/activated in equal quantities [26]. As such ATF6α & β are not competing in an antagonistic sense but the ratio of active α & β determines the level of response from a “full-on” α-rich activity to a much lower level but still positive β-rich response. If ATF6β were a true “antagonist” the, latter response would be total inhibition.

### ATF6 and novel treatment strategies for MCDS

All three canonical signalling pathways of UPR (IRE1, PERK, and ATF6) were shown to be activated in response to the increased ER stress in MCDS [6,7]. More recently, it was shown that the role of IRE1/*XBP1* branch of UPR in pathology of MCDS is redundant and non-essential [9]. While the physiological consequence of PERK ablation in MCDS remained to be identified, our results presented above show that ATF6 signalling has a key and indispensable role in MCDS pathology. Our results reveal a chondroprotective role for ATF6α and a much broader than anticipated role for ATF6β in the pathology of MCDS.

Although targeting and enhancing the ATF6α-dependent pathways and/or attenuating or blocking the ATF6β-dependent pathways represent possible novel avenues for future pursuit in terms of MCDS therapy, translation into potential therapies for MCDS would be challenging. Firstly, initial attempts to selectively modulate the ATF6 arm of the UPR, unlike PERK and IRE1, were unsuccessful largely due to the lack of identifiable small molecule binding sites on ATF6 [[44], [45], [46]]. Secondly, the great degree of homology between ATF6α and -β, further adds to the complexity of developing isoform selective modulators. More importantly, management of ER stress in vivo in the growth plate proved more complex than initially anticipated, as the clear beneficial effect of ATF6β ablation in hypertrophic chondrocytes was nullified by the detrimental effect on the adjacent proliferative chondrocytes. Therefore, a selective blocking of ATF6β would need to be specifically targeted to hypertrophic, whilst sparing proliferative chondrocytes to avoid adverse effects on proliferation rate.

Recently, we showed that treatment with carbamazepine (CBZ) enhanced the degradation of intercellular mutant collagen X proteins, alleviating the ER stress and reducing the disease severity in our MCDS mouse model [28]. Coupling CBZ administration with, for instance, an ATF6α-activity enhancing drug may prove an even more effective therapy for such ER-stress driven diseases.

## Materials and methods

### Ethics statement

The animal experiments performed in this study were approved by the University of Manchester Animal Welfare and Ethical Review Body. Animals were maintained, handled, and sacrificed in strict accordance with United Kingdom Home Office regulations.

### Transient transfection of HeLa cells with collagen X constructs

The wild-type, and mutants expression constructs encoding MCDS causing mutations in the full-length collagen X (N671K, G618V, Y598D, and NC1del10) were as described previously [30]. HeLa cells were cultured in Dulbecco's Modified Eagle's Medium (DMEM) (Sigma, D6429) supplemented with 10% foetal bovine serum (FBS) (Life Technologies, 10,500-064), penicillin (0.5 U/ml)/streptomycin (0.5 μg/ml) (Sigma, P0781) and 1% non- essential amino acid solution (Sigma, M7145) at 37 °C with 5% CO2. 24 h prior to transfection, cells were passaged and seeded at an appropriate density to achieve 70% confluency at the time of transfection. Cells were transiently transacted with 2 μg of collagen X expression construct DNA using Lipofectamine 3000 (Life Technologies, L3000) according to manufactures instructions. Cells were incubated at 37 °C for 24 h. Cell lysates were then harvested and further analysed with qPCR or SDS-PAGE and western blotting.

### siRNA-mediated gene silencing

A combination of two silencer® Select pre-designed and validated small interfering RNAs (siRNAs) (life technologies) were used to knockdown the expression of each gene of interest. For Atf6α: Atf6α Silencer® Select pre-designed siRNA (Life Technologies, s22688 & s22690) and for Atf6β Silencer® Select pre-designed siRNA (Life Technologies, s223330 & s3500). To achieve the best results HeLa cells were first reverse transfected with either 20 nM of Atf6α or 10 nM of Atf6β siRNAs duplexes using Lipofectamine RNAiMAX reagent (Life Technologies, 13,778) according to manufactures protocol and incubated for 24 h at 37 °C. The following day media was removed and cells were washed in PBS. siRNA-lipofectamine RNAiMAX complexes were prepared as previous and added drop-wise to the cells followed by overnight incubation at 37 °C. After a total of 48 h, cell lysate was harvested and knockdown efficiency was assessed at mRNA and protein levels with qPCR and SDS-PAGE western blotting, respectively.

### Co-transfection with Plasmid DNA and siRNAs

For co-transfection with plasmid DNA and siRNAs, HeLa cells were first reverse transfected with optimal concentrations of siRNA duplexes and RNAiMAX lipofectamine as described above and incubated for 24 h at 37 °C. The following day, media was removed and cells were washed in PBS. Plasmid DNA-lipofectamine 3000 complexes and siRNA-lipofectamine 3000 complexes, were prepared as described above and added drop-wise to the cells. Cells were incubated at 37 °C for a further 24 h. After total of 48 h, cell lysates were harvested and analysed by qPCR and SDS-PAGE western blotting.

### Harvesting protein samples from cells

Cell medium was discarded and cell layers were washed in 1× PBS. PBS was then removed and 50 μl 2× SDS-PAGE sample buffer contained a PhosSTOP phosphatase inhibitor cocktail (Roche, 04906845001) and a complete mini-protease inhibitor (Roche, 11836170001) and 10 μM MG132 proteasomal inhibitor (Sigma, M7449) was added to the each well of a 6-well plate. Cells were scarped using a cell scarper and placed into a 1.5 ml tube. Samples were heated for 10 min at 99 °C and then centrifuged at 13,000 rpm for 10 min at 4 °C. The lysate supernatant was transferred into a fresh 1.5 ml tube and the concentration of protein in the lysate was calculated using BCA assay (Thermo Scientific # 23227) with a bovine serum albumin standard curve according to manufacturer's protocol.

### SDS-PAGE and western blotting

40 μg of protein per sample for Atf6α protein detection and 20 μg of protein for all other proteins were loaded into the precaste NuPAGE® Novex® 4–12% Bis-Tris Gels (Life technologies, NP0322BOX). The gel was electroblotted onto a nitrocellulose membrane, which was blocked 1 h at room temperature with 5% skimmed milk powder in PBS containing 0.1% tween-20 and 2% (v/v) serum derived from the same species in which the secondary antibody was produced. The membranes were incubated in 1/500 dilution of Grp78/BiP (Santa Cruz, SC-1051), CRELD2 (Santa Cruz, sc-86110), and ATF6α (37–1) (Cosmo Bio Co, 73-505EX) primary antibodies and 1/1000 dilution of ATF6α (1–7) (BAM-73-500-EX), anti-ATF4 (Cell Signalling, 11815), Mouse IgG1 anti-His clone # (AD1.1.10) (R & D, MAB050), and GAPDH (L−20) (Santa Cruz, sc-31,915) primary antibodies in blocking solution overnight at 4 °C. Polyclonal rabbit anti-ATF6β antibody was produced as described previously [11]. It was diluted 1/333 in blocking solution overnight at 4 °C.

Membranes were then incubated with an appropriate HRP conjugated secondary antibody. An ECL detection kit (Life Technologies) and ECL hyperfilm (GE Healthcare) was used to develop the blots according to manufacturer's protocol.

The blots were quantified by densitometry analysis using ImageJ software on images from scanned films within the linear range of exposure. The intensity of each band was calculated relative to a loading control and standardised against a control protein sample on each blot. The result were analysed by ANOVA for statistical significance using GraphPad Prism 6.0 software.

### RT-qPCR analysis

Purification of RNA from cell lysate and homogenised ribs growth plate extracts in TRIzol reagent (Life Technologies) was performed using phenol and chloroform methodology. cDNA was synthesised from 1 μg of purified and DNase treated (Ambion, AM1906) RNA samples using the Taqman Reverse Transcription Reagent Kit (Applied Biosystems UK, N8080234) according to manufacturer's protocol. The following primers were used for real time qPCR:

ATF6α forward 5′-CACAGCTCCCTAATCACGTGG-3′ and reverse 5′-ACTGGGCTA TTCGCTGAAGG, ATF6β forward 5′-CAGCCATCAGCCACAACAAG-3′ and reverse 5′-GGCATCACCAGGGACATCTT-3′, CRELD2 forward 5′-GGGCTGGTGGACAAGTTTAAC-3′ and reverse 5′-CGAATCTCGCTGGACTCGTA-3′, ARMET forward 5′-TCACATTCTCACCAGCCACT-3′ and reverse 5′-TCACATTCTCAC CAGCCACT-3′, BiP forward 5′-GCTAATGCTTATGGCCTGGA-3′ and reverse 5′-CGCTGGTCAAAGTCTTCTCC-3′, CHOP human forward 5′-GCGCATGAAGGAGAAAGAAC-3′ and reverse 5′-TCTGGGAAAGGTGGGTAGTG-3′, CHOP mouse forward 5′-CCACCACACCTGAAAGCAGAA-3′ and reverse 5′-AGG TGCCCCCAATTTCATCT, spliced XBP1 forward 5′-GAAGCCAAGGGGAATGAAGT-3′ and reverse 5′-CCAGAATGCCCAACAGGATA-3′,and β-actin forward 5′-CCACCATGTACCCAGGCATT-3′ and reverse 5′-CACATCTGCTGGAAGGTGGA-3.

Each reaction was performed in duplicate using a StepOnePlusTM Real-Time PCR system (Life Technologies, 4376600). A no-template control was used along with other samples to check for contamination. The data generated from the real time PCR was analysed by ANOVA for statistical significance using GraphPad Prism 6.0 software.

Quantification of the mRNA level of the spliced form of XBP1 was performed as previously described [47]. Briefly, the synthesised single- stranded cDNAs were subjected to an initial 4 cycles of PCR to make double-stranded cDNAs as follow: 95 °C for 3 min, 4 cycles of 95 °C for 40 s, 60 °C for 45 s and 72 °C for 40 s, and then 10 min at 72 °C. In order to eliminate the unspliced form of XBP1 (XBP1U), which contains a *Pst*I restriction site within its sequence [47], the double-stranded cDNA mix was digested with 1.25 μl of *Pst*I restriction enzyme overnight at 37 °C. Subsequently the samples containing only spliced form of XBP1 (XBP1s) were subjected to a quantitative real time PCR as describe above.

### Generation of *Atf6*α^−^^/^^−^/ MCDS and *Atf6*β^−^^/^^−^/MCDS mice

*Col10a1* p.Asn 617Lys mice (ColX p.N617Km/m) [7] were crossed with mice that were knockout either for *Atf6*α or *Atf6*β [2] to generate the compound mutants, *Atf*α^−^^/^^−^/*Col10a1* p.N617Km/m and *Atf6*β^−^^/^^−^/*Col10a1* p.N617Km/m (*ATF6*α^−^^/^^−^/MCDS and *ATF6*β^−^^/^^−^/MCDS respectively). These mice were viable, fertile, bred normally and were handled in accordance with the Home Office Scientific Procedure Act (1986) guidelines. Genotyping was performed as previously described [7,18].

### Skeletal analysis

Mice at 3, 6, and 9 weeks of age were radiographed using a Flaxitron X-ray specimen radiography system (Flaxitron MX-20) and X-ray hyperfilm (GE Healthcare, GZ28906850). The lengths of femur and tibia bones and inner canthal distances (ICD) were measured from scanned radiographic images. All measurements were analysed by ANOVA for statistical significance using GraphPad Prism 6.0 software.

### Histology

The hind limbs were dissected, fixed and processed as previously described [28] prior to histological staining, immunohistochemistry or in situ hybridisation.

### H & E staining

H + E staining was performed as described previously [7] and all images taken using a Carl Zeiss Axiovision microscope fitted with an Axiocam colour CCD camera and associated Axiovision software.

Growth plate zone widths were measured on images of known magnification as described previously [7]. The height of PZ was defined from the point where round resting chondrocytes align into columns and become disc shaped to the point where HZ starts. The start of HZ was defined as proliferative chondrocytes stop proliferation, round up and become larger. The vascular invasion front was defined as the end point of HZ. For each animal, five slides at least 75 μm apart were measured and averaged. Measurements were analysed for statistical significance by ANOVA using GraphPad Prism 6.0 software. To measure heights of the most terminal hypertrophic chondrocytes (HCs) which is the height parallel to the direction of growth, four H&E stained slides per animal, at least 75 μm apart, were scanned using a digital slide scanner (Pannoramic 250 Flash, 3DHISTECH) and then analysed by an associated panoramic viewer software. For each tissue section, heights of one hundred hypertrophic chondrocytes located at the vicinity of vascular invasion front (the last three rows) were measured and averaged. The average height of the most terminal HCs for each animal was defined as an overall average between all related slides. All data were analysed by one-way ANOVA for statistical significance.

### Immunohistochemistry (IHC)

Immunohistochemistry for collagen X, BiP, and CRELD2 was performed on acetic acid/ethanol fixed tibial growth plate sections using the following primary antibodies; collagen X (polyclonal rabbit anti-collagen X against recombinant mouse NC1 domain), anti-Grp 78 goat polyclonal (Santa Cruz, SC-1051), and CRELD2 (R & D system, H3884) as previously described [7,48,49].

### Tartrate-resistant acid phosphatase (TRAP) staining

Osteoclasts at the vascular invasion front were stained using a TRAP staining kit (Sigma- Aldrich 387A) according to manufacturer's instructions. The number of positively stained cells (red/brown) were quantified per mm of vascular invasion front as described previously [28] in 3 sections per animal spaced 50 μm apart in 3 animals per genotype and analysed by one-way ANOVA for statistical significance.

### Bromodeoxyuridine (BrdU) labelling

BrdU labelling and analysis was performed as previously described [7]. At least three animals per genotype were included and analysed by one-way ANOVA for statistical significance.

### Terminal transferase dUTP Nick End Labelling (TUNEL) assay

TUNEL assay was performed on tibial growth plate sections of three-week old mice using a fluorometric TUNEL kit (Promega, G3250) according to manufacturer's protocol as describe previously [49].The slides were mounted with Vectashield with DAPI (Vector Laboratories, H1200) and imaged using the CoolSNAP ES Olympus BX51 camera and associated Metaview software. In order to obtain a reliable estimate of number of apoptotic hypertrophic cells in the growth plate, the green fluorescent apoptotic cells within the HZ were counted in three slides, at least 50 μm apart, per mouse and five animals for each genotype. The total number of hypertrophic chondrocytes within the HZ was defined by the number of DAPI-stained nuclei (as determined using ImageJ software). The apoptosis rate was defined as a percentage of the total number of hypertrophic cells within HZ. All data were analysed by one-way ANOVA for statistical significance.

### In situ hybridisation (ISH)

DIG-labelled colourimetric ISH was performed as previously described [7]. The resulting cDNA probes were cloned into pT7T3, linearised and transcribed using the appropriate restriction enzyme and RNA polymerase.

### Ribs growth plate extraction for use in western blotting

Protein extracts from ribs growth plate for western blotting were obtained as described previously [7,49]. Dissected growth plates from at least 3 mice were pooled together and placed either in 100 μl 2× SDS-PAGE sample buffer for western blotting or in 500 μl of TRIzol (Life Technologies, 15596018) for RNA extraction. Dissected growth plates were then homogenised using a micro-dismembranator. For western blotting, the homogenised rib growth plate extracts were boiled in SDS loading buffer containing β mercaptoethanol and centrifuged. The supernatant was collected and total protein concentration was assayed using the Pierce bicinchoninic acid (BCA) protein assay (Thermo Scientific # 23227) with a bovine serum albumin standard curve according to manufacturer's protocol. Protein extracts wee then analysed by SDS-PAGE and western blotting.

### PCR analysis to detect Xbp1 splicing in mouse rib extracts

Detection of *Xbp1* splicing on the rib growth plates from 3 week old mice were carried out as described previously [49]. 1 μg purified rib total RNA was reverse transcribed to cDNA using the Taqman Reverse Transcription Reagent Kit (Life Technologies) according to manufacturer's protocol and subjected to PCR with primers flanking the Xbp1 ER stress-responsive splice site F: 5′-GAACCAGGAGTTAAGAACACG-3′ and R: 5′-AGGCAACAGTGTCAGAGTCC-3′. PCR products, Xbp1U with the size of 205 bp and Xbp1s with the size of 179 bp, were then separated on 3% agarose gel. The level of Xbp1 splicing was then calculated as a percentage of the total Xbp1 (Xbp1s + Xbp1 U) using Image J software.

The following are the supplementary data related to this article.

**S1 Fig:**
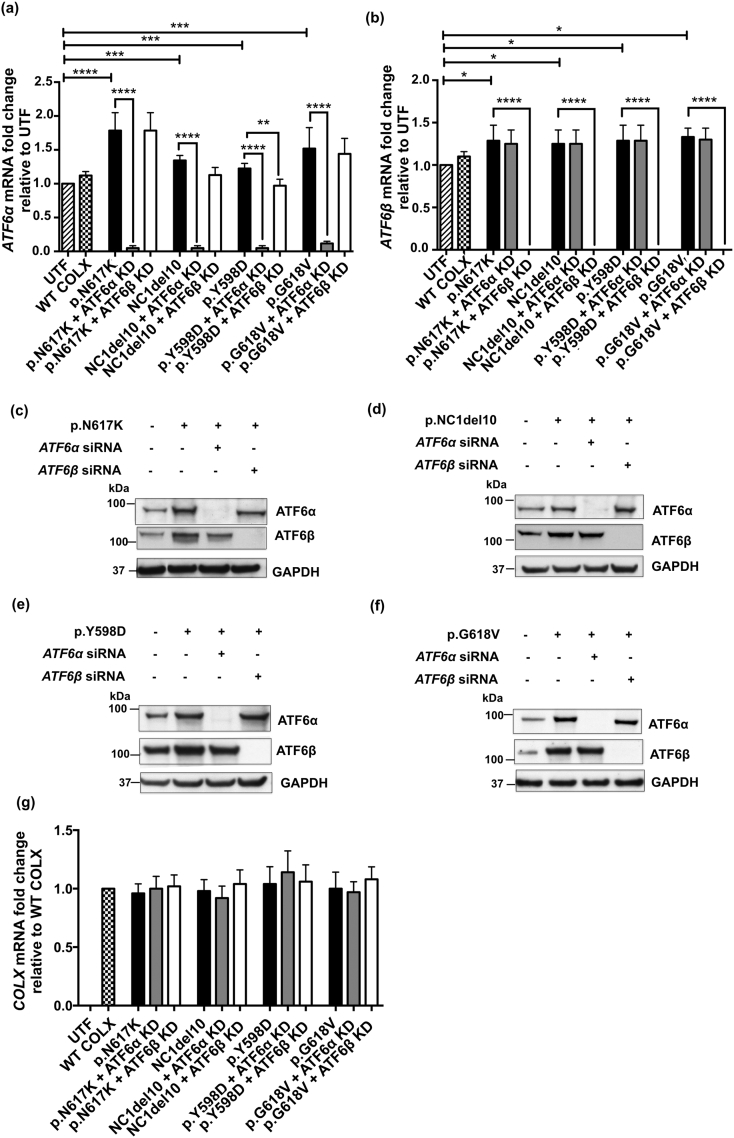
Confirming efficiency and specificity of knockdown for *ATF6*α and *ATF6*β in cells expressing different MCDS-causing mutant forms of collagen X protein. The expression of either ATF6α or ATF6β was knocked down in HeLa cells using siRNAs. The siRNA-mediated ATF6α or ATF6β knockdown cells were then transiently transfected with expression constructs encoding either the wild type collagen X or one of the following four MCDS-causing mutant forms of the protein: p.N617K, p.G618V, p.Y598D, and NC1del10. 48 h post transfection, RNA was extracted and analysed with real time qPCR for the expression of (a) *ATF6*α, (b) *ATF6*β*and* (*g*) *COL10A1*. The level of *ATF6*α and *ATF6*β mRNAs relative to β-actin was normalised against the untransfected cells. The level of *COL10A1* mRNA relative to β-actin was normalised against the cells expressing the wild type collagen X. Mean ± SEM (*n* = 5). *p < 0.05, **p < 0.01, ***p < 0.001, ****p < 0.0001. All data were analysed for statistical significance by ANOVA. Typical western blots for ATF6α and ATF6β proteins in ATF6α or ATF6β siRNA-mediated knocked down cells expressing (c) N671 K (d) NC1del10, (e) Y598D, and (f) G618 V mutants forms of collagen X.

**S2 Fig:**
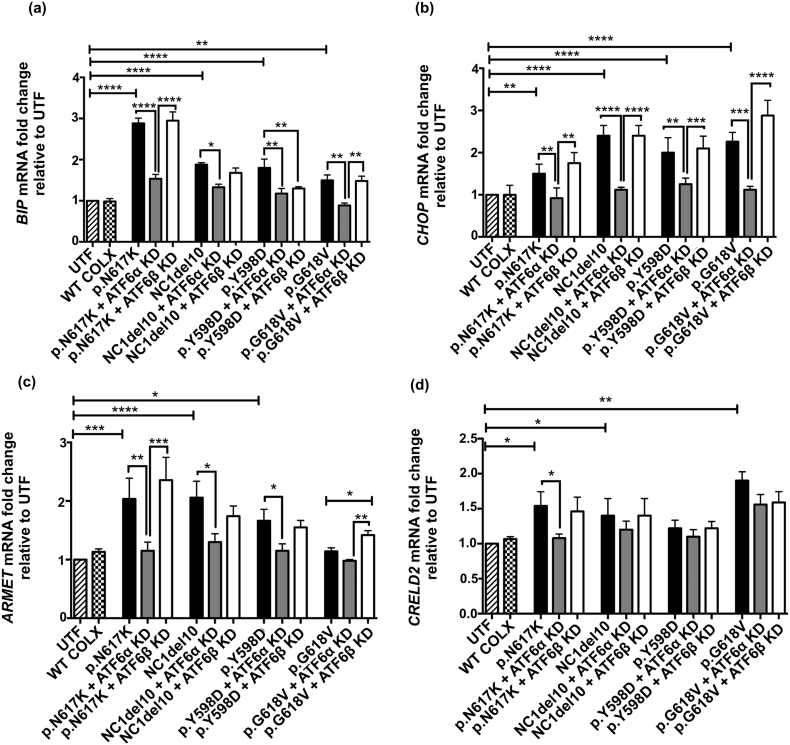
Effects of *ATF6*α and *ATF6*β knockdown on general markers of ER stress in cells expressing different MCDS-causing mutant forms of collagen X protein. The expression of either *ATF6*α or *ATF6*β was knocked down in HeLa cells using siRNAs. The siRNA-mediated *ATF6*α or *ATF6*β knockdown cells were then transiently transfected with expression constructs encoding either the wild type collagen X or one of the following four MCDS-causing mutant forms of the protein: p.N617K, p.G618V, p.Y598D, and NC1del10. 24 h after transfection of the MCDS constructs, cell lysates were extracted and analysed with qPCR for mRNA of (a) *BIP*, (b) CHOP, (c) ARMET and (d) CRELD2. Mean ± SEM (n = 5). *p < 0.05, **p < 0.01, ***p < 0.001, ****p < 0.0001. Untransfected cells (UTF) served as controls.

**S3 Fig:**
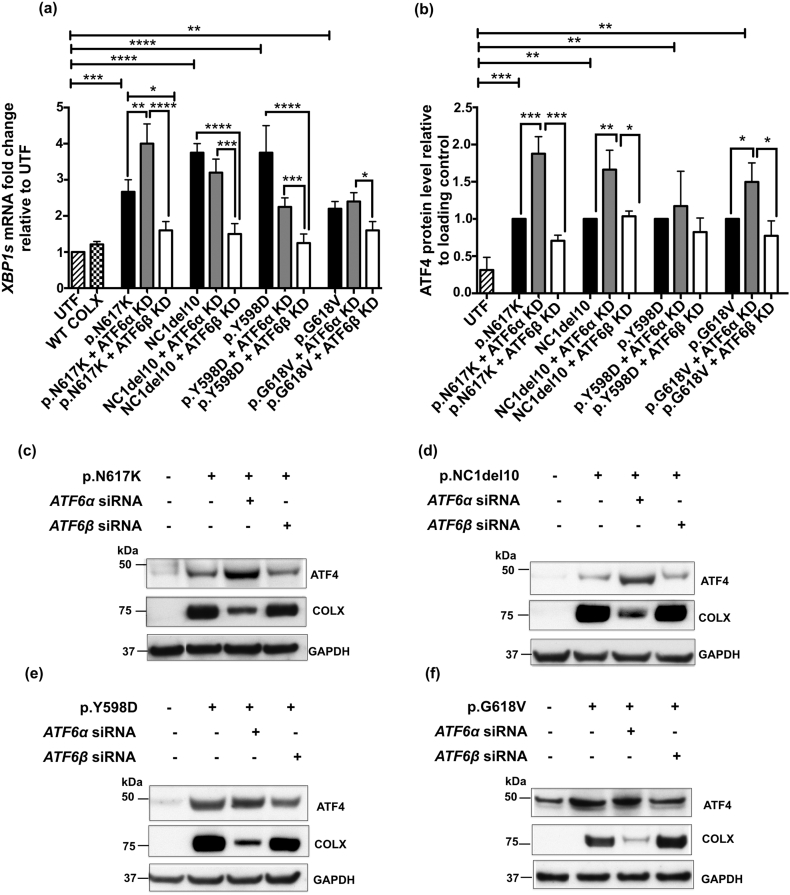
ATF6 independent measure of ER stress induced by four different MCDS-causing mutations in cells that were knocked down either for *ATF6*α or *ATF6*β. The expression of either *ATF6*α or *ATF6*β was knocked-down in HeLa cells using siRNAs. These cells were then transiently transfected with expression constructs encoding either the wild type collagen X or one of the four MCDS-causing mutant forms of the protein: p.N617K, p.G618V, p.Y598D, and NC1del10. 24 h after transfection of the MCDS constructs (a) RNA was extracted and analysed with real time qPCR for the expression of the spliced *XBP1*, *XBP1s*. The level of *XBP1s* relative to β-actin was normalised against the untransfected cells. Mean ± SEM (n = 5); and (b) cell lysates were prepared and immunoblotted using an anti- ATF4 antibody. The level of ATF4 protein relative to GAPDH loading control was standardised to the cells expressing the same type of MCDS-causing collagen X mutation. Values represent Mean ± SEM from three independent experiments. *p < 0.05, **p < 0.01, ***p < 0.001, ****p < 0.0001 as determined by ANOVA. UTF: Untransfected cells. Typical western blots for ATF4 and collagen X proteins in *ATF6*α or *ATF6*β siRNA-mediated knocked down cells expressing (c) N671 K (d) NC1del10, (e) Y598D, and (f) G618 V mutants forms of collagen X.

**S4 Fig:**
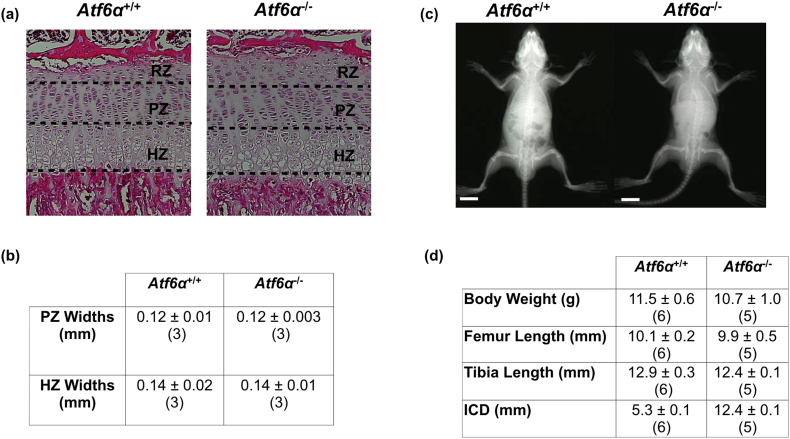
Characterisation of three week old mice that were knockout for *Atf6*α. (a) H & E staining of tibial growth plate from mice with specified genotype at three weeks of age (RZ = resting zone, PZ = Proliferative zone, and HZ = hypertrophic zone). (b) Measurement of widths of proliferative and hypertrophic zones. Mean ± SEM (N). (c) A typical X-ray image from three week old mice that were either wild type or knockout for *Atf6*α. White scale bar = 100 μm (f). Body measurements at three weeks of age. Mean ± SEM (N).

**S5 Fig:**
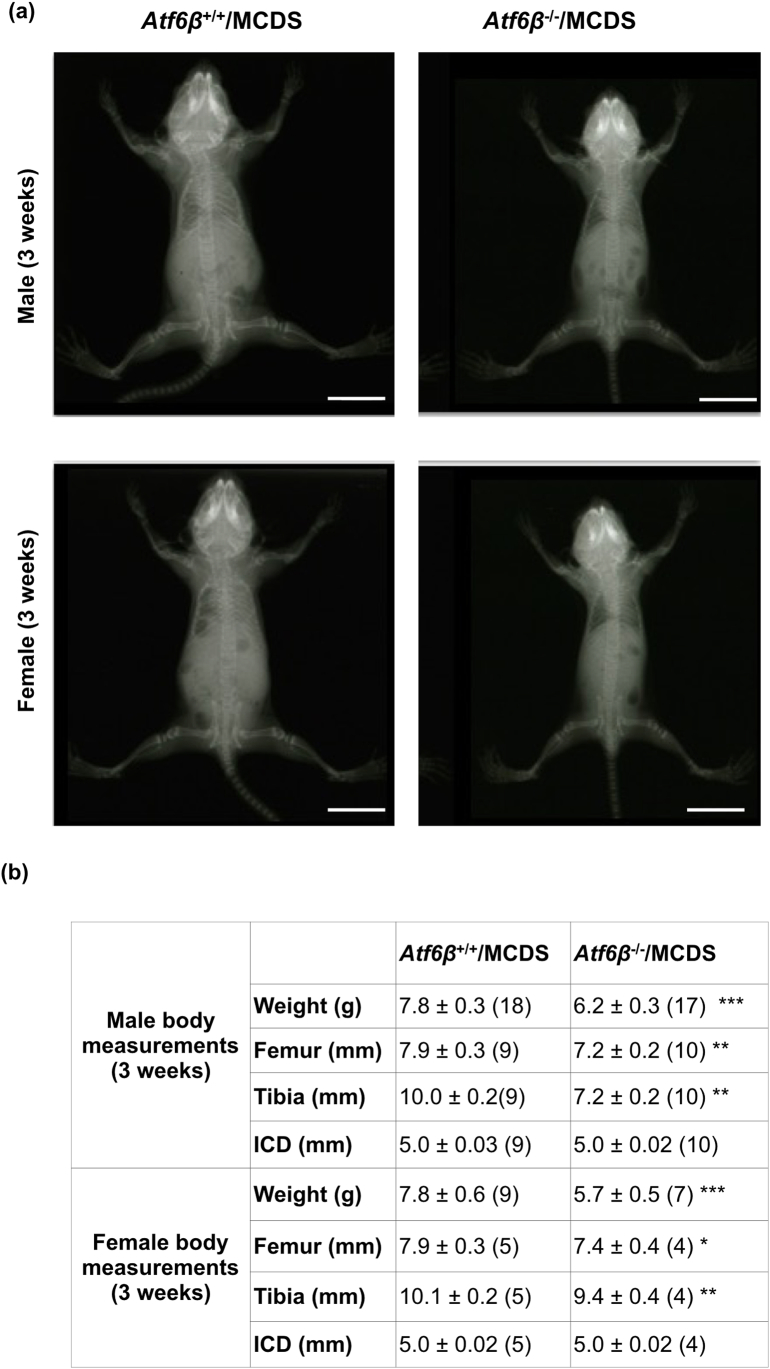
Effects of *Atf6*β ablation on the skeletal development and body weight of three week old MCDS mice. (a) A representative X-ray radiograph image for three-week old male (top row) and female (bottom row) MCDS mice with the specified genotypes. White scale bar = 100 μm (b) Mean ± SEM (N). *p < 0.05,**p < 0.01, and ***p < 0.001 when compared to *Atf6*β+/+/MCDS. All statistical analysis by ANOVA.

**S6 Fig:**
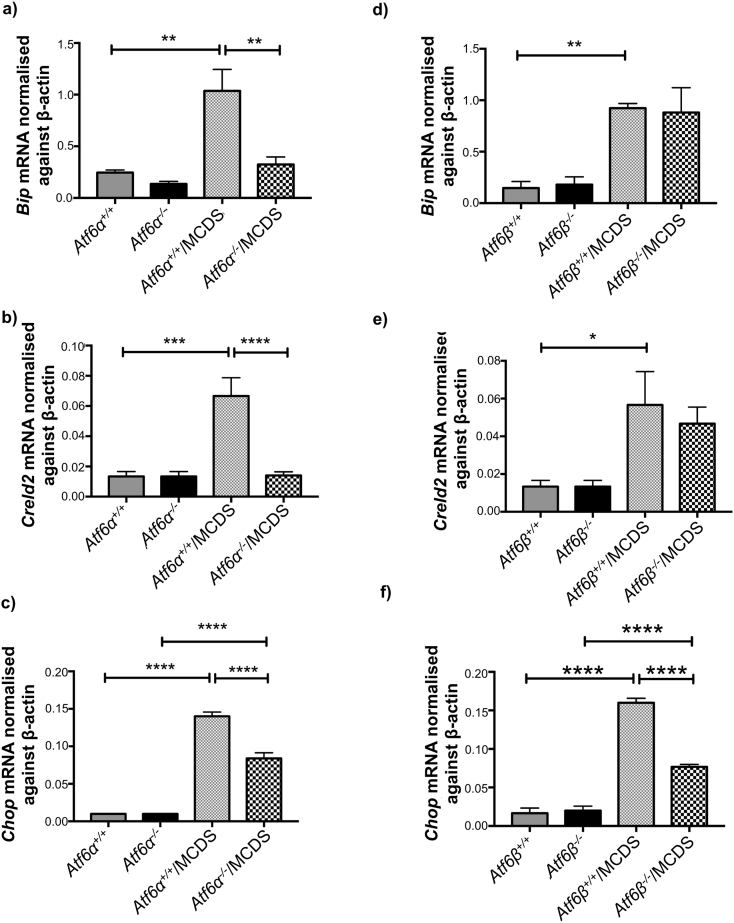
Analysis of mouse ribs growth plate extracts for the expression of *Bip*, *Chop*, and *Creld2* mRNAs in the absence of Atf6α or *Atf6*β. Total RNA from at least two pooled ribs growth plate extracts of 21-day old mice were extracted and analysed with qPCR for mRNA of (a & d) *Bip*, (b & e) *Chop*, and (c & f) *Creld2*. Mean ± SEM (*n* ≥ 3). *p < 0.05, **p < 0.01, ***p < 0.001, ****p < 0.0001.
